# CXCR4 inhibition with AMD3100 attenuates amphetamine induced locomotor activity in adolescent Long Evans male rats

**DOI:** 10.1371/journal.pone.0247707

**Published:** 2021-03-01

**Authors:** Briana Mason, Corey Calhoun, Victoria Woytowicz, Latifa Pina, Roshninder Kanda, Curtis Dunn, Antonio Alves, S. Tiffany Donaldson

**Affiliations:** 1 Department of Pharmacology, University of Texas Health Science Center at San Antonio, San Antonio, Texas, United States of America; 2 Department of Psychology, University of Massachusetts Boston, Boston, Massachusetts, United States of America; University of Kentucky, UNITED STATES

## Abstract

Adolescent psychostimulant abuse has been on the rise over the past decade. This trend has demonstrable ramifications on adolescent behavior and brain morphology, increasing risk for development of addiction during adolescence and in later adulthood. Neuroimmune substrates are implicated in the etiology of substance use disorders. To add to this body of work, the current study was developed to explore the role of a chemokine receptor, CXC Chemokine Receptor 4 (CXCR4), in the development of amphetamine (AMPH) sensitization. We targeted CXCR4 as it is implicated in developmental processes, dopaminergic transmission, neuroimmune responses, and the potentiation of psychostimulant abuse pathology. To evaluate the role of CXCR4 activity on the development of AMPH sensitization, a CXCR4 antagonist (Plerixafor; AMD3100) was administered to rats as a pretreatment variable. Specifically, adolescent Long Evans male rats (*N* = 37) were divided into four groups: (1) AMD3100 (IP, 4.0 mg/kg) + AMPH (IP, 4.0 mg/kg), (2) saline (SAL; 0.9% NaCl) + AMPH, (3) AMD3100 + SAL, and (4) SAL + SAL. Animals were first habituated to locomotor activity (LMA) chambers, then injected with a pretreatment drug (AMD3100 or SAL) followed by AMPH or SAL every other for four days. After a one-week withdrawal period, all animals were administered a low challenge dose of AMPH (IP, 1.0 mg/kg). AMPH-injected rats displayed significantly more locomotor activity compared to controls across all testing days. CXCR4 antagonism significantly attenuated AMPH-induced locomotor activity. On challenge day, AMD3100 pre-treated animals exhibited diminutive AMPH-induced locomotor activity compared to SAL pre-treated animals. Postmortem analyses of brain tissue revealed elevated CXCR4 protein levels in the striatum of all experimental groups. Our results implicate CXCR4 signaling in the development of AMPH sensitization and may represent an important therapeutic target for future research in psychostimulant abuse.

## Introduction

Psychostimulant drug abuse during adolescence potentiates the risk for progression to drug dependence in adulthood for human [1, 2] and rodent [3, 4] populations. Psychostimulant abuse remains a pervasive and significant issue for adolescents that may rise within the next decade [5, 6]. Preclinical and clinical models implicate adolescence, compared to adulthood, as a vulnerable developmental period for the development of psychostimulant-induced neurobehavioral alterations [1, 7, 8, as reviewed by 9]. Psychiatric and substance use disorders can be explored in validated animal models [as reviewed by 10] to investigate the biological substrates that underlie psychostimulant abuse vulnerability during adolescence to further aid the development of viable pharmacotherapies.

A novel line of scientific inquiry operationalizes psychostimulants as activators of neuroimmune signaling mechanisms that affect neuroplasticity and drug-seeking behavior in preclinical models [as reviewed by 11], including adolescent paradigms [as reviewed by 12, 13]. Several rodent *in vivo* and *in vitro* models have shown that drug classes such as alcohol [14, 15] and opioids [16–19] enhance neuroinflammatory signaling through increased activity of chemoattractant cytokines (“chemokines”). CXC Chemokine Receptor 4 (CXCR4) is a G-protein coupled receptor that induces and propagates significant proinflammatory signaling cascades following activation by its only ligand, CXC Chemokine Ligand 12 (CXCL12, stromal derived factor 1; SDF-1) [20, as reviewed by 21]. CXCR4 was first discovered *in vitro* as a co-receptor for human immunodeficiency virus infection, and, when antagonized, prevents viral replication [22, 23]. In rodent and human neural *in vitro* [24] and *in vivo* models [25], CXCR4 may determine cell fate by activating secondary messengers like extracellular signal-regulated kinases 1/2 and Jun N-terminal kinase to reduce cyclic adenosine monophosphate and increase intracellular calcium levels. These activities ultimately lower the threshold for action potentials, altering neuronal and glial signaling. CXCR4 has been associated with the onset of several diseases such as Alzheimer’s and Parkinson’s diseases, chronic pain, and the development of various cancers [25, as reviewed by 26–31]. CXCR4 antagonism reduces disease symptomatology [32–35]. Similarly, CXCR4 antagonism has been shown to reduce the characteristic neurobehavioral patterns of dysregulation associated with the reinforcing effects of stimulant drugs [36]. Kim and colleagues [37] showed that CXCR4 antagonism prior to an acute administration of cocaine prevented increased locomotor activity and disrupted the cocaine-induced conditioned place preference. Clinical evidence from abstinent lifetime cocaine users implicates CXCL12-CXCR4 and other proinflammatory substrates as predictors of cocaine symptom severity and suggests the use of these substrates as biomarkers for the development of intervention protocols for psychostimulant use disorders and psychiatric comorbidities [38]. Furthermore, clinical and rodent models demonstrate anti-inflammatory agents as effective therapies for psychiatric and substance use disorders [as reviewed by 39, 40].

Although stimulants affect multiple monoamine systems, actions on dopamine receptors in human and rodent populations are critical [as reviewed by 41]. Psychostimulants like cocaine can cause long-term damage to the human and rodent brain by inducing severe neurotoxicity to cells, altering the overall rate of enzymatic activity, and disrupting dopamine release in neural regions associated with reward [42–44]. Dopaminergic neurons originate in the ventral tegmental area (VTA) and substantia nigra (SN) and project to the nucleus accumbens (NAc), striatum (caudate putamen/CPu in animal models), frontal cortex, and regions that are collectively defined within the limbic system [as reviewed in 45] and use dopamine and its receptors to communicate throughout the circuit in the mammalian brain [46, 47].

Modulation of dopaminergic activity in the mesocorticolimbic system is requisite for general psychostimulant seeking behavior and the reinforcing effects of psychostimulants in mammals [48–50]. We build upon this preclinical and clinical evidence and hypothesize that CXCR4 may play a role in psychostimulant-addiction like behavior. CXCR4 is expressed on the plasma membrane surface of dopaminergic neurons, microglia, and astrocytes in the mammalian brain [51, 52]. Psychostimulant exposure affects dopaminergic and neuroimmune substrates in the mammalian striatum [as reviewed by 53, 54]. Furthermore, rodent models report evidence that implicates dopamine receptor activity as a regulator of nervous system immune activity in other psychiatric and physiological pathologies [55, as reviewed in 56]. The CXCL12-CXCR4 axis interacts with neurons to influence synaptic pruning and growth in rodent and *in vitro* models [57, as reviewed in 58], neurotrophic factors in rodent and clinical models [59, 60], and rodent hippocampal neurogenesis [61].The hippocampus is of interest as dopamine transmission affects hippocampal inputs to the striatum *in vitro* [62] and goal-directed behavior in a rat model [63]. During rodent nervous system development, CXCR4 may influence adolescent response to stimulants since CXCR4 binding to CXCL12 and subsequent signaling activity induces progenitor glial cell migration to layers of the early cortex and the hippocampus in the initial organization of the brain [64–66] and cultured human neural precursor cells [67]. While many facets of CXCR4’s developmental role are unexplored, it has been shown that amygdalar *CXCR4* expression remains upregulated in adulthood, following an initial adolescent exposure to cocaine [68]. Collectively, these data suggest the adolescent brain may be differentially affected by psychostimulant-induced alterations in neuroimmune signaling and thus, confers a persisting vulnerability that increases the risk for progression to addiction in adulthood.

It is, therefore, probable that CXCL12-CXCR4 dysregulation may be implicated in the effects of repeated psychostimulant use and arguably influences adolescent vulnerability to addiction-like behaviors. The present study examined the role of CXCR4 antagonism on the development of AMPH sensitization in a cohort of adolescent male Long Evans rats. We hypothesized that pretreatment with a CXCR4 receptor antagonist, the bicyclam drug AMD3100 (Plerixafor; 1,19-[1,4-phenylenebis(methylene)]-bis1,4,8,11-azatetradecane), would affect the development of AMPH-induced locomotor sensitization. Additionally, we measured CXCR4 protein levels using immunohistochemistry to determine if differences in striatal CXCR4 expression mapped onto differential AMPH-induced locomotor behaviors.

## Materials and methods

### Ethics

All animal experiments and listed protocols were conducted in accordance with guidelines established by and approved by the Institutional Animal Care and Use Committee (IACUC) at the University of Massachusetts Boston. Procedures detailed here also followed the applicable portions of the Animal Welfare Act and the National Institute of Health’s ‘Guide for the Care and Use of Laboratory Animals’ (NIH Publications No. 80–23; Revised 1996).

### Experimental subjects

A total of 40 adolescent (purchased on postnatal day [PND] 32–34, 200–350 g) male Long Evans rats were ordered from Charles River Breeding Laboratories (Wilmington, MA, United States). A power analysis to determine the number of animals required to detect significant differences between groups, was calculated using G*Power software [69] and was based on past analyses performed in our laboratory [70, 71]. Statistical significance was set to p ≤ 0.05, and *β* = 0.80. After arrival, animals were housed in groups of 2–3 in ventilated cages (229.90 × 82.50 × 81.06 cm) (Lab Products; Seaford, DE, United States) with 3.85–4.00 cm of contact bedding. Animals were randomly sorted into experimental groups by cage (SAL-SAL, SAL-AMPH, AMD-SAL, AMD3100 (Plerixafor)-AMPH, *n =* 9–10 per group) and were maintained in a temperature- and humidity-controlled environment with food and water accessible *ad libitum*. Animals were maintained on a 12:12 h light/dark cycle, and lights were on from 0700 h to 1900 h. Testing did not begin until after animals were habituated to the facility for 10 days (PND 42–44). All testing was performed during the light cycle between 1200–1600 h.

### Drug pretreatment and four-day amphetamine sensitization regimen

Every other day for four days, animals were brought into the testing room and allowed to habituate for 30 minutes. Subjects were weighed and then placed into one of four locomotor activity chambers. Locomotor activity (LMA) testing was performed as previously described in our laboratory [72]. LMA chambers were directly connected to a computer running MedAssociates locomotion tracking software (MedAssociates, St. Albans, VT, United States). Each commercial LMA chamber (dimensions: 17 × 17 × 12 cm) was equipped with photoelectric beams to record distance traveled, stereotypies, and rears when photobeam transmissions were broken by an animal subject. Stimulants are known to induce persistent locomotor movements that are classically considered as consequences of increased dopamine activation to the striatum. Distance traveled was defined by each rat’s individual gait and their movement from one point to another. Stereotypies were defined as head bobbing, twitching, licking, or biting that were captured as rapid movements between photobeams. Rearing behavior was defined as the animal raising up above typical height and breaking the vertical photobeam transmission. Animals were placed in the LMA chamber for 15 minutes prior to pretreatment to obtain a baseline measurement of typical LMA across each of these three dependent variables. After baseline observations, LMA recordings were paused and pretreatment injections were intraperitoneally (IP) administered as appropriate to group: isotonic saline (0.9% sterile isotonic saline/SAL) or AMD3100 (4.0 mg/kg, dissolved in saline; Plerixafor/CXCR4 antagonist; MedChem Express, Monmouth Junction, NJ, United States). Animals were then returned to their respective chamber and the LMA tracking software was resumed. At the 30 min time point, recording software was paused again and animals were injected with either SAL or AMPH 4.0 mg/kg D-amphetamine sulfate; Sigma, St. Louis, MO, United States). Following drug injections, animals were returned for a final 60 min recording for a total of 90 min for the testing period. Between testing periods, LMA data were extracted and the LMA chambers were cleaned between each set of experimental subjects with mild soap and water. This was repeated every 48 h for four days after which all animals were given a 7-day withdrawal period. Finally, animals were challenged with a low dose of AMPH (1 mg/kg, IP), sacrificed, and brain tissue was harvested and stored at -80°C for later histological analyses.

### Challenge dose

**Fig 1:**
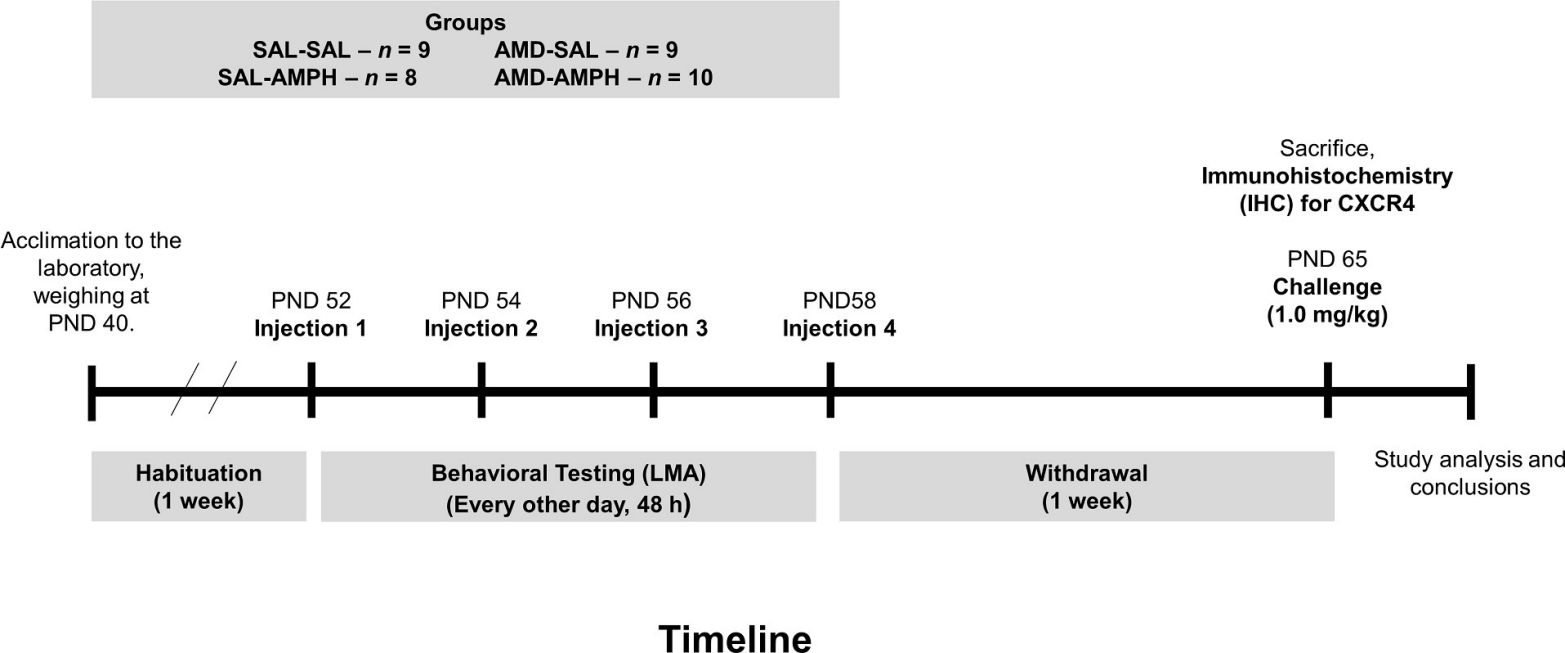
Timeline that depicts for the experimental design and procedures used in the current study. Animals were habituated to the laboratory setting for one week prior to the start of testing. Following this, animals were placed in the LMA chambers for a 15 min habituation, and then given a pretreatment (SAL, 0.9% NaCl, IP) or (AMD,4.0 mg/kg, IP). This was followed by drug treatment, (SAL or AMPH 4.0 mg/kg, IP) at 30 min, every 48 h for four days. One week after the final day of testing, which was the withdrawal period, all animals were challenged with a low dose of AMPH (1.0 mg/kg, IP: timepoint 30 min) after a 30 min habituation in the LMA.

One week following the termination of the four-day AMPH sensitization regimen, all animals were retrieved from their cages, weighed, and brought to the LMA testing room where they were habituated to one of four LMA chambers for 30 min. Animals were injected with a challenge dose of AMPH (IP, 1.0 mg/kg) and tested for an additional 60 min, for a total of 90 min. Data were extracted from MedAssociates^TM^ tracking software after testing completion. Animals were sacrificed via live decapitation and brain tissue was immediately harvested and stored in -80°C for later immunohistochemical analyses. The comprehensive timeline for this experiment can be seen in **Fig 1.**

### Euthanasia and brain extraction

Following the final AMPH challenge and subsequent LMA assessment, animals were removed from the LMA chamber and prepped for sacrifice. Animals were placed inside decapicones (Braintree Scientific, Braintree, MA; United States) and were promptly sacrificed via guillotine. Upon decapitation, brains were extracted and snap frozen on dry ice and stored at -80°C in a freezer until microsectioning was performed for later immunohistochemical analyses.

### Immunohistochemistry for CXCR4

At the time of histology, brains were removed from the -80°C freezer, embedded in freezing O.C.T. compound (mounting medium), and adhered to a metal chuck for frozen (-20°C) microsectioning in a cryostat (Leica CM 3050S; Leica Biosystems, Welzlar, Germany). Brains were blocked and 30-μm coronal sections were taken to target the dorsal striatum. Next, sections were mounted on frosted glass sides (Fisherbrand Superfrost Plus; ThermoScientific, Waltham, MA, United States). Anatomical assessment and location of the caudate putamen was determined using a rat brain atlas [73]. The slides were thoroughly covered and post-fixed in 2 mL of 4% paraformaldehyde for 30 min. Slides were rinsed, and then cryoprotected by dousing in incremental sucrose-paraformaldehyde solutions (5% - 20%). Next, slides were gently agitated in 0.05M NaPBS repeatedly for 1 h, with the NaPBS solution changed every 20 min. Following this, slides were rinsed in H_2_O_2._ A mixture of 1.5% normal goat serum-NaPBS to block endogenous peroxidases, and after a 5 min NaPBS rinse, slides were incubated overnight at 4°C in primary anti-CXCR4 antibody (1:1000 in Triton-X and NaPBS; Abcam, Boston, MA; United States; Abcam Antibody Code ab2074).

On the next day, the slides were removed from the refrigerator and then rinsed in NaPBS and incubated in goat-anti-rabbit secondary antibody (1:600 in Triton-X and NaPBS; ABC Elite Kit, Vector Labs, Burlingame, CA, USA) at room temperature for 1 h. Following this, sections were rinsed again in NaPBS and then processed through an avidin-biotin complex (ABC) method (Vectastain ABC HRP kit; Denver, CO, United States) and processed in Trizma-based buffer (Sigma Aldrich; Natick, MA). Finally, the sections were stained with a 3,3’-diaminobenzidine (DAB) horseradish peroxidase (HRP) substrate kit (Vectastain ABC HRP kit; Denver, CO, United States) for 5 min, at which time the slides were again rinsed in Tris buffer to terminate the reaction. The slides were left to dry overnight under a ventilated fumehood with cover to protect from dust artifacts. The next day, the slides were rehydrated with increasing concentrations of ethanol and xylene, and then cover slipped with Permount for microscopy and subsequent image analysis. Negative controls were run following the exact protocol outlined above except for incubation in the primary antibody.

### Image analysis

Digital images of the stained slides were taken using light microscopy (Olympus BX-40; Pennsylvania, United States) fitted with a monochrome Scion Image camera and software (4× and 10× magnification). Magnification at 4× was used to confirm impregnation of neuronal and glial cells for all animals relative to the negative controls. Higher magnification images were then used for counting positive CXCR4 immunoreactive cells. Counts were made using CellTarget^TM^ with a threshold set to count the number of CXCR4 positive cells. When imaged at 4× magnification, experimenters blind to group representation were guided to count all the CXCR4 positive neurons visible in the dorsolateral, dorsomedial, and dorsoventral striatum. When imaged at 10× magnification, experimenters utilized the quadrant function of CellTarget^TM^ [74] to maintain consistent cell counts from sample to sample.

### Statistical analysis

All statistical analyses were completed using SPSS software (Windows and Mac, version 22.0). Data were first analyzed for normality using the Kolmogorov-Smirnov Test. To identify significant differences between groups, multivariate two-way analyses with repeated measures of variance (ANOVAs) were used. For behavioral data, three independent 2 (pretreatment) X 2 (drug treatment) repeated measures (Day and testing time block) mixed factor ANOVAs were employed to evaluate main and interaction effects across the independent variables for (1) distance traveled (2) rearing behavior and (3) stereotypies. To support these findings, we averaged total distance traveled (cm), stereotypies, or rears made by each subject across the four days and performed a univariate ANOVA on the average cumulative recorded movements for each behavioral measure for each subject. To identify AMPH-induced locomotor activation following a low-dose AMPH (IP, 1 mg/kg) challenge after a one-week withdrawal period, 2 (pretreatment) X 2 (drug treatment) mixed factors were utilized to identify main and interaction effects for each dependent measure. All behavioral data were reported as group means ± SEM. For neural data, a 2 (pretreatment) X 2 (drug treatment) mixed factor ANOVA was employed to evaluate main and interaction effects for CXCR4 positive cells in the dorsal striatum. Three experimenters who were blinded to experimental conditions and showed 0.90 inter-rater reliability quantified immunohistochemistry data. Immunohistochemistry data are reported as group means ± SEM. All significant findings were interpreted using Bonferroni correction and Tukey’s Honest Significant Difference (HSD) post-hoc tests.

## Results

Three subjects were excluded from analyses due to low weight gain, data loss, and, in the case of one animal, escape from the LMA chamber (*N* = 37, *n* = 8–10 subjects per group). There were no significant differences in weight in the remaining subjects when measured (**S1 Fig**). We analyzed locomotor behavior (distance traveled, rears, and stereotypies) extracted from MedAssociates LMA software using a 2 (pretreatment) X 2 (drug treatment) mixed factors ANOVA f with repeated measures for Day and testing time period.

Data were analyzed for normality using the Kolmogorov-Smirnov test of normality. On the first [D(19) = 0.260; *p* = 0.001], second [D(19) = 0.237, *p* = 0.006], and fourth day of testing [D(19) = 0.289, *p* = 0.0001], the data violated normality assumptions. Data from animals in the antagonist group also violated the test for normality on the first and [D(19) = 0.302, *p* = 0.0001] and fourth [D(19) = 0.203, *p* = 0.039] days of testing. We elected to retain the data for analysis due to (1) retention of normality in the habituation period on all four days of sensitization testing, (2) positive skewness towards zero as a result of low distance traveled and related movements, and (3) no more than two outliers becoming apparent per group.

### AMD3100 reduces AMPH-induced distance traveled, stereotypies, and rears

#### Distance traveled

In the analyses performed, we observed no significant differences for Day [F_3,31_ = 1.562, *p* = 0.218] or Time Period [F_1,33_ = 0.553, *p* = 0.462] alone on distance traveled. All animals moved around the locomotor activity chamber over the progression of testing, and this movement increased each day [Day × Time Period, F_3,31_ = 7.424, *p* = 0.001 < 0.05, η_p_^2^ = 0.418]. There were no significant interaction effects for Day × Time Period × Pretreatment [F_3,31_ = 2.268, *p* = 0.100 > 0.05] or Day × Time Period × Pretreatment × Treatment [F_3,31_ = 1.229, *p* = 0.316 > 0.05]. Pretreatment with AMD3100 alone did not produce any change in rat behavior as indicated by non-significant results for Day × Pretreatment [F_3,31_ = 1.692, *p* > 0.05].

**Fig 2:**
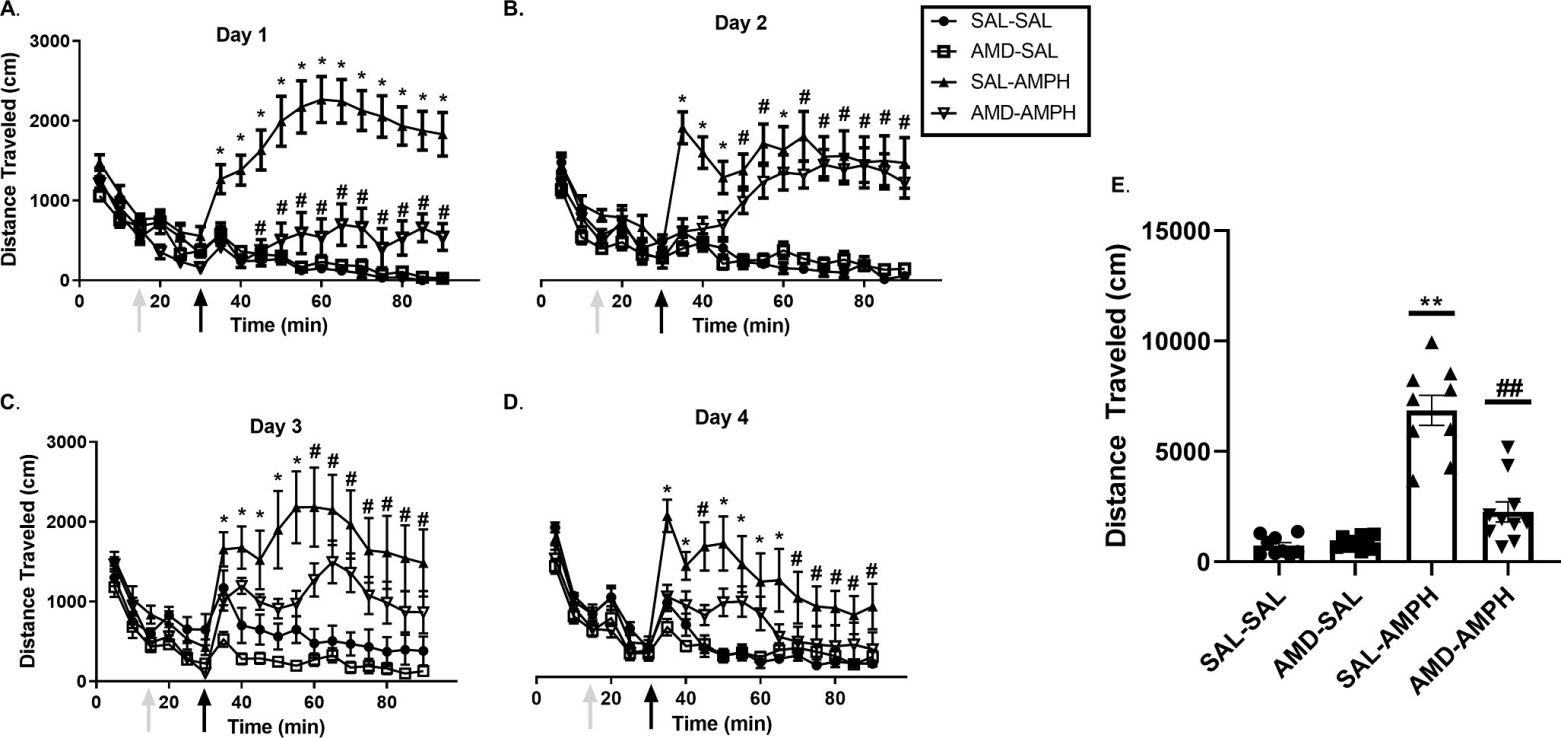
The locomotor effects of repeated pretreatment and treatment schedule on all experimental groups.

**Fig 3:**
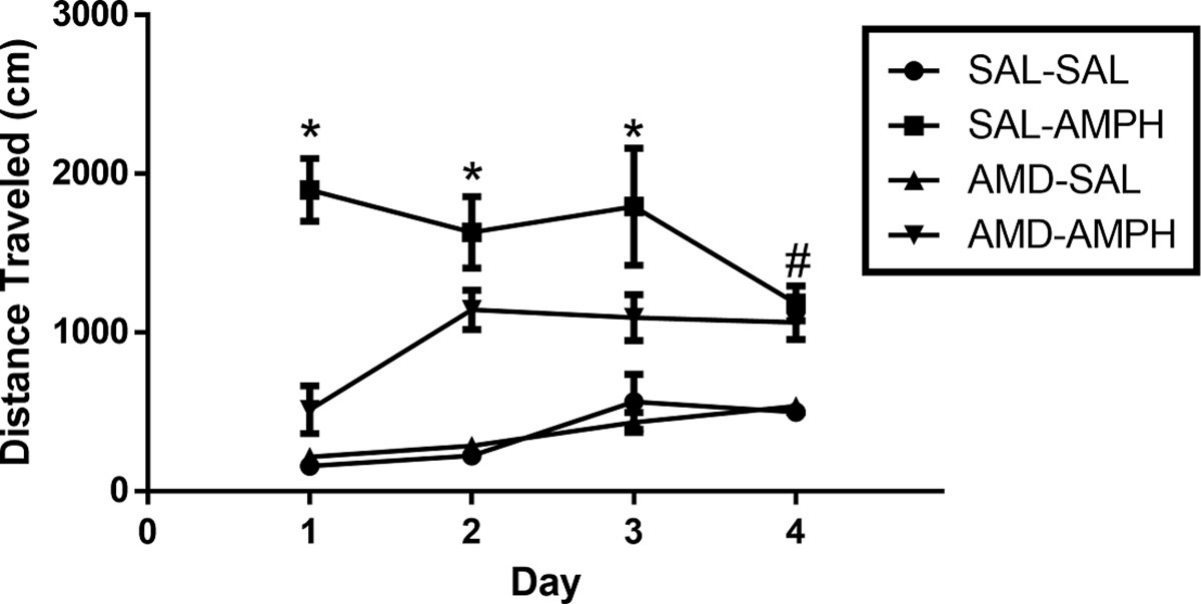
The effect of pretreatment and treatment conditions on average cumulative distance traveled (cm) across the four days of testing and on challenge.

The two-way ANOVA did reveal significant interaction effects of Day × Treatment [F_3, 31_ = 4.917, *p* = 0.007 < 0.05, η_p_^2^ = 0.322] and Day × Pretreatment × Treatment [F_3,31_ = 4.046, *p* = 0.015 < 0.05, η_p_^2^ = 0.281]. Rats in the SAL-AMPH group traveled further than their drug-naïve counterparts in the SAL-SAL group. Additionally, AMD3100-AMPH animals traveled less than SAL-AMPH animals overall, especially on the first day of testing. Furthermore, AMD3100-AMPH animals exhibited significantly greater LMA activity compared to SAL-SAL and AMD-SAL animals (**Fig 2**). We supplemented our data analysis by calculating the percent change (distance traveled from 30 to 90 min *minus* distance traveled from 0–30 min) for each subject across the four main days of testing to account for general individual differences and treatment effects. We observed a significant effect of Treatment for Day 1 [F_1,32_ = 39.501, *p* < 0.001, η_p_^2^ = 0.552], Day 2 [F_1,32_ = 43.030, *p* < 0.001, η_p_^2^ = 0.574], Day 3 [F_1,32_ = 14.738, *p* < 0.001, η_p_^2^ = 0.315], and Day 4 [F_1,32_ = 16.237, *p* < 0.001, η_p_^2^ = 0.337]. These findings indicated that AMPH treatment caused a significant increase in the distance traveled for adolescent rats regardless of pretreatment (**Fig 3**).

Locomotor activity was measured for 90 min (data represented as Mean ± SEM). Arrows indicate an initial 0–15 min habituation period to the LMA chamber, followed by (1) the pretreatment injection period 15–30 min, (2) the post-injection 30–90 min period on (A) Day 1, (B) Day 2, (C) Day 3, and (D) Day 4. Single asterisks (*) represent a significant difference compared to all other groups (*p* < 0.05), and single hash marks (#) represent a significant differences compared to SAL-SAL and AMD-SAL groups (*p* < 0.05). (E) Data are presented as a scatterplot, with bars indicating average distance traveled (cm) in the Treatment testing period (30–90 minutes) ± SEM. Double asterisks (**) and double hash marks (##) represent significant differences between labeled groups and all other groups (*p* < 0.01).

Effect of pretreatment and drug treatment on the average distance traveled (cm) across each of the 4 days of testing (Mean ± SEM). Asterisks (*) represent significant differences (*p* < 0.05) as compared to other groups, and a hash mark (#) represents significant differences (*p* < 0.05) collapsed between AMD-AMPH and SAL-AMPH groups as compared to the following control groups, SAL-SAL and AMD-SAL.

#### Stereotypies

**Fig 4:**
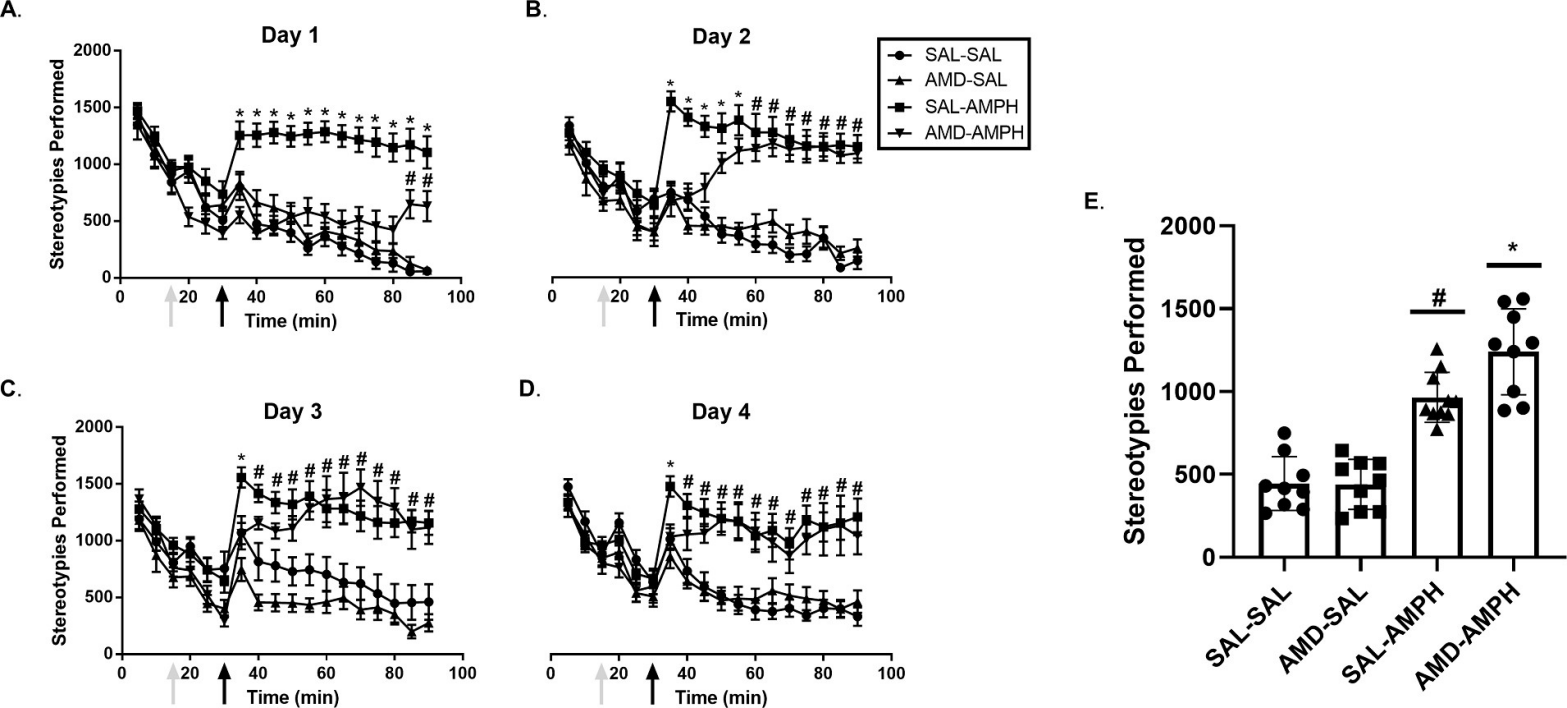
Average stereotypies performed following pretreatment and treatment conditions over the four-day period. Average stereotypies over the 4-day period following Pretreatment (AMD or SAL, grey arrows at time point 15 min) and Treatment (SAL or AMPH, black arrows at time point 30 min) conditions on (A) Day 1, (B) Day 2, (C) Day 3, and (D) Day 4. After observing main and interaction effects, post-hoc analyses identified significant pairwise groups differences. Asterisks (*) represent significant differences between the SAL-AMPH relative to all other groups. Hash marks (#) represent significant differences between the AMD-AMPH and all other groups. (E) The data are represented as a scatterplot of the cumulative average of stereotypies completed by each group over the four days of testing (Mean ± SEM).

We report significant main and interaction effects of Day [F_3,31_ = 6.716, *p* = 0.001 < 0.05, η_p_^2^ = 0.394], Day × Time Period [F_3,31_ = 14.688, *p* = 0.0001 < 0.05, η_p_^2^ = 0.587] and Day × Time Period × Pretreatment [F_3,31_ = 3.151, *p* = 0.039 < 0.05, η_p_^2^ = 0.234] for stereotypies. There was also a significant interaction effect of Time Period × Pretreatment × Treatment [F_1,33_ = 4.947, *p* = 0.033 < 0.05, η_p_^2^ = 0.130], demonstrating the combined effect of repeated testing on overall stereotypies (**Fig 4**). Although total stereotypies made over the four individual days were not significantly different for Pretreatment × Treatment, [F_1,132_ = 1.608, *p* = 0.207 > 0.05, n.s.], there was a significant interaction effect of Day × Pretreatment × Treatment [F_3_,_132_ = 3.272, *p* = 0.023 < 0.05, η_p_^2^ = 0.069]. SAL-AMPH treated rats engaged in significantly more stereotypies across the total 4 days as compared to all other groups, where AMD-AMPH animals exhibited diminished amounts of stereotypies compared to SAL-AMPH animals on Day 1.

#### Rears

**Fig 5:**
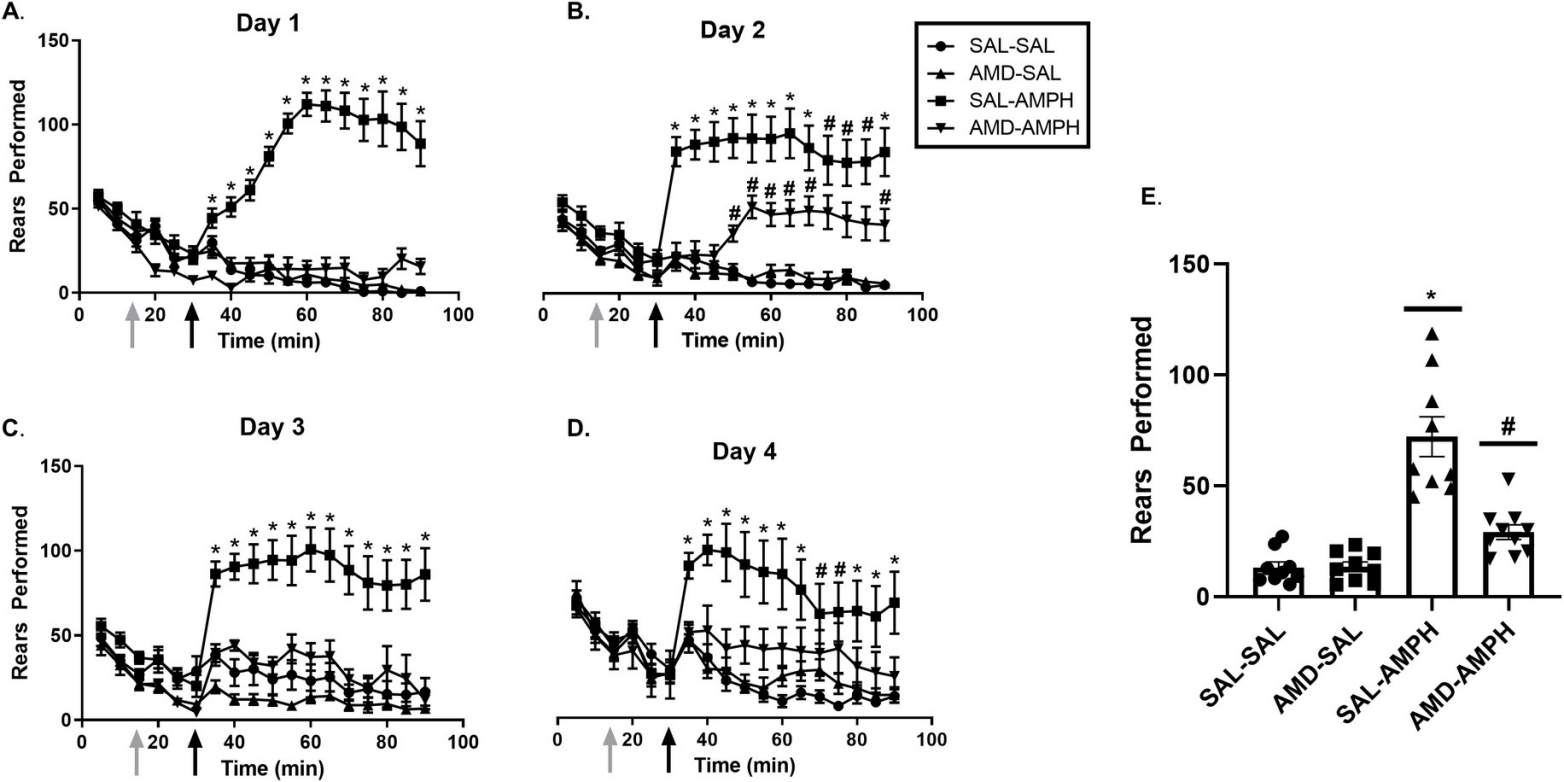
The effects of the treatment conditions on rears performed over the four days of testing. Data are represented as Mean ± SEM. Cumulative average vertical counts (Mean ± SEM) performed over the four -day (A-D) testing period. (E) The mean of rears for each group over the four days of testing. Asterisks (*) represent significant differences between the SAL-AMPH group and all other groups (*p* < 0.05), and hash marks (#) indicate significant differences between the AMD-AMPH and SAL-SAL, AMD-SAL, and SAL-AMPH groups (*p* < 0.05).

There were no significant group differences for rears that were identified in the analysis of the habituation period (*p* < 0.05) on each day of the 4-day sensitization regimen. For all other testing periods, data analysis revealed that SAL-AMPH animals reared significantly more than all other groups across all four days of testing, and this was confirmed through a Tukey HSD test with significance set at *p* < 0.05. Furthermore, AMD3100-AMPH animals were not significantly different from SAL-SAL or AMD3100-SAL groups in terms of cumulative rears on Day 1 of testing (*p* < 0.05). In the remaining experimental days, multivariate analysis revealed a significant effect of Day × Pretreatment [F_3,31_ = 9.808, *p* = 0.002 < 0.05, η_p_^2^ = 0.371] as well as an interaction effect of Day × Pretreatment × Treatment [F_3,31_ = 7.624, *p* = 0.001 < 0.05, η_p_^2^ = 0.425]. Tukey HSD post-hoc test indicated that SAL-AMPH animals reared the most as indicated by an interaction of Day × Treatment effect [F_8,26_ = 9.493, *p* = 0.0001 < 0.05, η_p_^2^ = 0.723], and AMD3100-AMPH animals reared significantly more than SAL-SAL and AMD3100-SAL groups but significantly less than the SAL-AMPH group (*p* < 0.05) (**Fig 5**).

### AMD3100 pretreatment reduces AMPH sensitization following a one-week drug-free period

**Fig 6:**
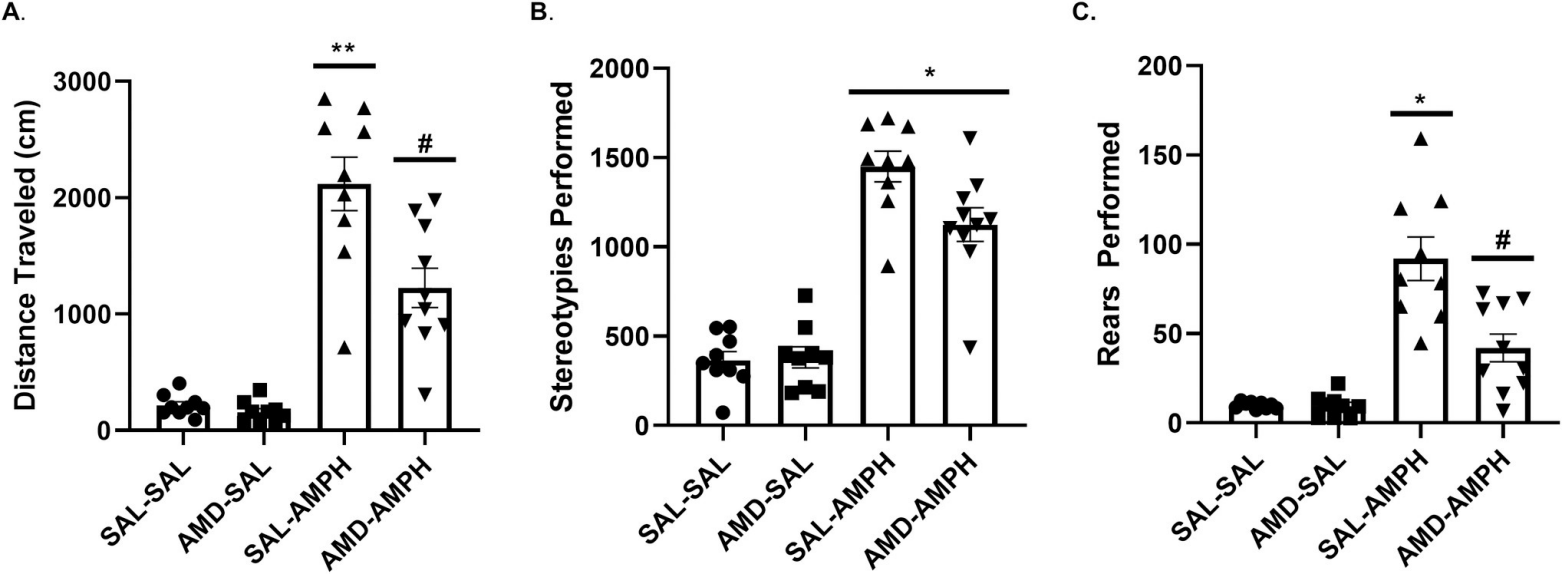
Distance traveled, stereotypies, and rears observed after a 1.0 mg/kg challenge dose of AMPH one week following the four-day AMPH sensitization regimen. Data are represented as Mean (A) distance traveled (B) stereotypies, and (C) rears performed ± SEM. Asterisks (*) represent statistically significant differences set at *p* < 0.05 as compared to AMD-SAL and SAL-SAL groups, double asterisks represent statistically significant differences at *p* < 0.01, and hash marks (#) represent statistically significant differences between AMD-AMPH and all other groups at *p* < 0.05.

After a one week drug withdrawal period from behavioral experimentation, all animals were challenged with a low dose of AMPH (1.0 mg/kg, IP) to test the expression of AMPH sensitization. Two-way ANOVA analysis revealed significant main effects of pretreatment [F_2,32_ *=* 5.237, *p* = 0.01 < 0.05, η_p_^2^ = 0.247] and treatment [F_2,33_ = 54.263, *p* = 0.001 < 0.05, η_p_^2^ = 0.772] on total distance traveled. We also report a significant interaction effect of Pretreatment × Treatment [F_2,32_ = 4.133, *p* = 0.025 < 0.05, η_p_^2^ = 0.205] (**Fig 6A**). A Tukey post-hoc HSD test (*p* < 0.05) revealed that animals pretreated with AMD3100 exhibited locomotor responses to a low dose AMPH challenge (IP, 1mg/kg) similarly to that of SAL pretreated animals. However, the AMD3100-AMPH group traveled significantly less in response to low dose AMPH challenge compared to SAL-AMPH animals. Past pretreatment with AMD3100 or SAL did not have an effect on overall stereotypies (Baseline: F[_2,33_ = 0.037, *p* = 0.848 > 0.05]; Treatment: F[_2,33_ = 4.077, *p* = 0.052 > 0.05]). We performed a Bonferroni correction on these data and again found that Pretreatment did not reach significance (*p* = 0.65 > 0.05). A significant main effect of Treatment [F_2,33_ = 143.452, *p* = 0.001 < 0.05, η_p_^2^ = 0.813] and an interaction effect of Pretreatment × Treatment [F_2,33_ = 5.001, *p* = 0.032 < 0.05, η_p_^2^ = 0.132] were found in our analyses. This indicated that AMD3100-AMPH and SAL-AMPH groups exhibited near-equivalent numbers of stereotypies in response to a low dose AMPH challenge (**Fig 6B**).

Behavioral rearing responses for the four groups were similar to the responses we observed for distance traveled. We found a significant main effect of Pretreatment [F_2,33_ = 5.730, p = 0.007 < 0.05, ηp^2^ = 0.205] as well as an interaction effect of Pretreatment × Treatment [F_2,32_ = 6.231, p = 0.005 < 0.05, ηp^2^ = 0.280]. We determined this to mean that the AMD-AMPH group showed significantly reduced rears relative to the SAL-AMPH group, but potentiated activity as compared to controls (SAL-SAL, AMD-SAL groups) that had never been treated with AMPH in the past. Animals in the control conditions (SAL-SAL, AMD-SAL) reared significantly less than AMD-AMPH and SAL-AMPH animals (p < 0.05), as identified through a Tukey HSD post-hoc test (**Fig 6C**).

### CXCR4 protein expression is upregulated following repeated AMPH exposure

**Fig 7:**
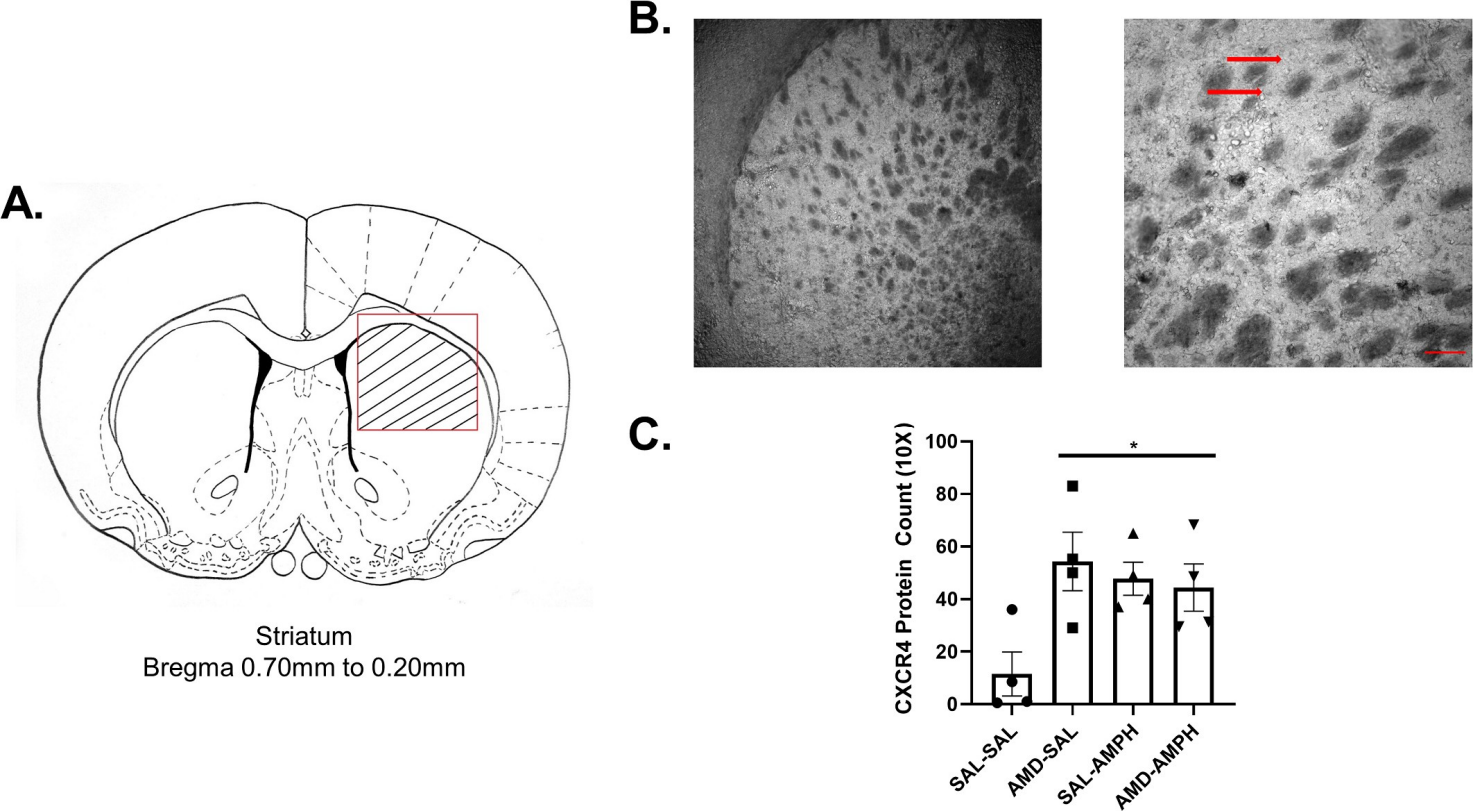
Repeated administration of the CXCR4 antagonist AMD3100 and D-amphetamine sulfate increased CXCR4 protein levels in the dorsomedial striatum. Striatal neurons and glia were visualized with Nickel-DAB after immunostaining for CXCR4, viewed, and imaged on a light microscope (N = 16, *n* = 4 per group). (A) Cells were counted specifically within the striated targeted region outlined in red on the line drawing, delineating the dorsal striatum. Bregma +0.70 mm to + 0.20 mm ranges were selected based on rat brain atlas coordinates [73]. (B) Sample staining at 4× magnification and 10× magnification. The scale bar on the bottom right represents ~240 pixels (154.8 um). The red arrows identify deeply stained striatal puncta and cellular processes. (C) All groups expressed CXCR4 within the dorsal striatum. These numbers were elevated across experimental groups relative to the control (data represented as Mean ± SEM), asterisks (*) represent significant differences set at *p*<0.05.

We investigated changes in dorsal striatal CXCR4 receptor protein levels following repeated SAL or AMPH exposure with or without AMD3100 pretreatment. Brain sections ranging from Bregma +0.20 mm to +0.70 mm were taken from *n* = 16 animals. Representative images of CXCR4 staining are shown in **Fig 7A and 7B)**. Pearson’s product-moment correlation coefficient assessed inter-rater reliability at 4× magnification [r = 0.693, *p* = 0.03] and 10× magnification [r = 0.697, *p* = 0.001]. Counts at 20× magnification were also correlated between independent researchers [r = 0.679, *p* = 0.05].

CXCR4 positive cell counts between all independent researchers were averaged for all subject tissues. A 2 (pretreatment) X 2 (drug treatment) mixed factor ANOVA revealed a significant main effect of Pretreatment [F_3,12_ = 6.774, p = 0.023 < 0.05]. Repeated exposure to AMD3100 pretreatment upregulated the number of CXCR4 positive cells and clusters or puncta in the dorsal striatum of AMD3100-AMPH and AMD3100-SAL animals (**Fig 7C**).

## Discussion

The current study evaluated the role of CXCR4 signaling in the development of AMPH-induced locomotor sensitization in adolescent male Long Evans rats. We antagonized CXCR4 receptor protein prior to repeated AMPH treatment to determine if it would interfere with the development of AMPH sensitization. Our findings indicate that pretreatment with the CXCR4 antagonist, AMD3100, interferes with the development of AMPH-induced locomotor sensitization and attenuates the sensitized AMPH response to a low dose challenge following a one-week drug withdrawal period. Accordingly, CXCR4 protein levels in the dorsomedial striatum were significantly elevated in response to repeated AMPH treatment for both levels of the pretreatment independent variable (AMD3100 and SAL), directly linking the effects of repeated AMPH exposure to dorsal striatal neuroimmune function *in vivo*.

We have previously shown that a low to moderate dose of amphetamine (3.0 mg/kg) is sufficient to induce sensitization and neuronal GABAergic alterations in a sexually dimorphic manner, where control and ovariectomized females were more locomotive and hyperactive than males [75]. We adapted this behavioral sensitization model for the current experiment and found that AMD3100 pretreatment significantly reduced total distance traveled and rears, but not stereotypies, in adolescent male AMPH-treated rats. All AMPH-treated rats displayed an upregulation of CXCR4-immunopositive cells in the dorsomedial striatum with no differences for animals pretreated with AMD3100. Taken together, the data implicate CXCL12-CXCR4 activity in the development of behavioral sensitization to repeated AMPH administration. The present experimental data does not allow for a causal link between neuroimmune data and behavioral outcomes. However, due to the timing of our AMPH sensitization model it is possible that we missed acute effects of AMD3100 pretreatment on the neuroimmune axis. Thus, at the time of measurement we were unable to distinguish any differences in CXCR4 expression between the pretreatment groups. Further work is required to determine if CXCR4 and CXCL12 mRNA and protein levels vary as a function of day(s) of AMPH- or AMD3100-treatment across neural regions in the dopaminergic mesocorticolimbic circuit.

Chemokines are emerging as critical feedback and regulatory system messengers in response to the use of psychostimulant drugs. As chemokines become better elucidated in the literature, there is an emerging body of evidence that highlights the role of chemokine signaling in neuronal communication and thus, could be necessary for the development and expression of responses to psychostimulant drugs. For example, human in vitro models [76] implicate CC Chemokine Receptor 5 (CCR5) mechanisms in the cellular response to methamphetamine, while silencing genes for and blocking CC Chemokine Receptor 2 (CCR2) and CCR5 elicit an attenuation in conditioned place preference and locomotor behavior to methamphetamine or cocaine in rodent *in vivo* models [77–79]. We have likewise demonstrated that systemic CXCR4 antagonism attenuates AMPH-induced hyperlocomotion in adolescent male Long Evans rats. Indeed, CXCR4 is evidenced to be expressed throughout the rodent adolescent [51] and adult brains [80, 81]. Furthermore, chronic amphetamine exposure amplifies the excitability of dopaminergic and glutamatergic neurons in the rodent prefrontal cortex [82, 83], which is one of the last areas of the mammalian brain to fully mature structurally and functionally [84–90]. As a critical developmental period, adolescence confers a lasting vulnerability to structural and functional neurobehavioral changes in response to environmental challenges [as reviewed by 91] in human adolescents as compared to adults [92]. Additionally, in a cohort of male and female rats, it was found that the *timing* of repeated amphetamine exposure centered around the onset of puberty determined later susceptibility to anxiety-like behavior and modulated dopamine D1 receptor activity levels in the ventral striatum during AMPH withdrawal periods [93]. Similarly, psychostimulant-induced striatal fos expression is dependent upon dopamine D1 and D2 receptor activation in the rat brain [94]. The current study reports that CXCR4 antagonism during the development of AMPH-sensitization had a significant effect on the expression of AMPH-induced hyperlocomotion and significantly upregulated the expression of striatal CXCR4 protein expression following a terminal AMPH challenge. CXCR4 protein expression was also significantly upregulated in all AMPH-treated adolescent animals as compared to SAL-SAL controls, providing *in vivo* evidence of AMPH-induced neuroimmune alterations in the adolescent rat striatum. While the current study did not examine dopamine receptor expression in conjunction with striatal CXCR4 expression, future work should aim to further delineate the role of CXCL12-CXCR4 signaling in relation to dopamine receptor activity following repeated adolescent AMPH exposure.

Future work should also address sex differences, since modifications in impulsivity [95], vigilance [96], cognitive flexibility [97], memory [98], and an enhanced sensitivity to the effects of psychostimulants in adulthood are reported [as reviewed by 99 and 100] highlighting risk factors for the progression to addiction. Biological females are considered more sensitive to the effects of psychostimulants than males, and often exhibit exaggerated behavioral responses to psychostimulants that are regulated by circulating female hormone levels [101, as reviewed by 102]. In our future research, we intend to incorporate female animals to examine the relation between adolescence and sex as a biological variables in the development and expression of AMPH sensitization.

The decreased effectiveness of AMD3100 in our AMD-AMPH group over the four days of testing could represent the characteristic neuromolecular plasticity associated with adolescence and synaptogenesis within the CXCL12-CXCR4 axis. Accordingly, it is not surprising that AMD3100 pretreatment had no effect on the development of sensitized AMPH induced stereotypies. In their 1997 review, Pierce and Kalivas argue that repeated administration to psychostimulants can induce differential sensitization of ambulatory and stereotyped behavior [103]. Furthermore, exposure to stress and subsequent glucocorticoid receptor activation alters rodent locomotor behavioral sensitization in response to repeated psychostimulant administration [104, 105]. Adolescence also modulates sensitized behavioral responses to psychostimulant exposure in rodents, as a single exposure to a high dose of cocaine differentially induced behavioral sensitization to a challenge dose [106]. Taken together, the discrepancy in sensitized locomotor responses between ambulatory and stereotyped behaviors may be attributed to (1) rodent experimental differences in sensitized locomotor responses to repeated psychostimulant administration, (2) natural differences in trait-anxiety phenotypes and subsequent activation of the hypothalamic-pituitary-adrenal (HPA) axis, and (3) the adolescent developmental period conferring differentiated responses to repeated amphetamine administration in rodents. Thus, it is possible that these mechanisms are unique to the adolescent brain. The development and reorganization of the adolescent brain require greater immune and nutrient availability for growth that could be shifted by psychostimulant experience. The refinement and modification of neuronal and glial systems in the prefrontal cortex and throughout the rest of the brain, is aided in part by chemokines [107, as reviewed by 108]. The CXCL12-CXCR4 axis has specific functions to aid progenitor cell migration, proliferation, and axonal pathfinding in the neonatal period [66, 109–111]. Although CXCR4 levels decrease two weeks following the neonatal period, when they have reached their temporary maximal peak [112], we report evidence that repeated exposure to a moderate dose of AMPH significantly increased adolescent CXCR4 expression in the dorsomedial striatum. We, therefore, hypothesize that CXCR4 is recruited in the presence of repeated AMPH exposure to increase signaling, synaptic plasticity, and neuronal activation throughout the adolescent brain.

We also observed that AMD3100-SAL treated rats exhibited a significant increase in CXCR4 protein levels comparable to that of AMD3100-AMPH and SAL-AMPH treated rats. CXCR4 is downregulated in cortical circuit neurons from the early postnatal period throughout adolescence and into adulthood in mammals [66, as reviewed by 113]. In the present study, the observed changes in CXCR4 protein expression may be a compensatory attempt by the adolescent neuroimmune microenvironment to correct cell positioning and function after the neurotoxic effects of repeated AMD3100 and AMPH exposure on cell activity. Furthermore, it is also likely that given AMD3100 affects other signaling mechanisms, their recruitment may serve to augment CXCR4 protein levels. Another possibility is that any suppressive effects of AMD3100 on striatal CXCR4 expression may be acute and, therefore, our one-week withdrawal period is too long to detect transient changes that may have occurred during the four-day AMPH sensitization regimen. Future experiments should be designed to delineate these theoretical possibilities. Moreover, differential CXCR4 protein expression is evidenced in other psychiatric disorders [114] and physiological system dysregulation [115, 116] as well. Our findings demonstrate that striatal CXCR4 receptors are upregulated in response to repeated AMPH exposure and CXCR4 antagonism and these changes are linked to CXCL12-CXCR4 axis alterations observed in other diseased/disordered states.

Although we did not extend our findings to additional dopaminergic mesocorticolimbic regions that regulate psychostimulant addiction-like behaviors, there is an emerging body of literature that implicates other dopaminergic region alterations in chemokine activity and relates this to psychostimulant exposure related rodent behaviors [37, 77, 117–120]. For example, exogenous CXCL12 administration prior to cocaine injection into the nucleus accumbens shell has inhibitory effects on rat activity [120]. Furthermore, CXCL12 signaling mediates the migratory process and maintenance of *in vitro* and *in vivo* rodent dopaminergic neurons within the VTA [121] and CXCL12 protein levels are required for executive function and inhibitory gating within the mPFC [122, 123]. Thus, chemokine activity is implicated in psychostimulant-related behaviors throughout the dopaminergic mesocorticolimbic pathway. Future research should center on further elucidating the role of various chemokines systems in the mesocorticolimbic pathway on the rewarding and psychomotor-activating effects of psychostimulants.

One potential direction that could clarify the depth of CXCR4 activity on the development and maintenance of AMPH sensitization would be to better understand the genetic and epigenetic properties of CXCR4. In the present study, increased expression of striatal CXCR4 protein could be indicative of compensatory mechanisms for intracellular regulation, such as heterologous dimerization with CXCR7 or the production of upregulated proinflammatory signaling factors like nuclear factor kappa beta (NF-κB), toll-like receptor 4 (TLR4), and Protein kinase B that follow G-protein coupled receptor activation [96, 124, 125]. A future direction for our animal model would be to assess and map genetic and epigenetic substrates within these proinflammatory pathways to psychostimulant-induced behavioral outcomes. For example, repeated methamphetamine exposure upregulates mouse striatal CCR2 mRNA and increases expression of the epigenetic marker histone H3 lysine 4 (H3K4) trimethylation at the CCR2 promoter region [79]. Additionally, in a mouse model, paternal sire cocaine use affected filial 1 offspring’s drug preference along with significant chemokine (and other systems associated with psychostimulant exposure and neurodevelopment) and gene expression changes in the ventral striatum [126]. Drug-induced behavioral and epigenetic alterations to chemokine systems within the rodent mesocorticolimbic pathway extends to other drugs of abuse as well, such as morphine [127]. Taken together, genetic and epigenetic markers map onto psychostimulant exposure and chemokine activity in the rodent brain and this relation should be further explored in future work.

The present study demonstrates that CXCR4 antagonism with AMD3100 is sufficient to modify the development of amphetamine sensitization in a male adolescent rat model. We show here that dorsomedial striatal CXCR4 protein levels are enhanced by repeated AMPH exposure and CXCR4 antagonism. These findings further implicate the CXCL12-CXCR4 axis in the development of amphetamine sensitization and encourages additional experimental research to examine adolescent psychostimulant vulnerabilities and/or therapeutic development involving this neuroimmune axis.

## Supporting information

S1 Fig (TIF)

## References

[1] Chen CY, Storr CL, Anthony JC. Early-onset drug use and risk for drug dependence problems. Addictive Behaviors. 2009 3 1;34(3):319–22. 10.1016/j.addbeh.2008.10.021 19022584 PMC2677076

[2] Vida R, Brownlie EB, Beitchman JH, Adlaf EM, Atkinson L, Escobar M, et al. Emerging adult outcomes of adolescent psychiatric and substance use disorders. Addictive Behaviors. 2009 10 1;34(10):800–5. 10.1016/j.addbeh.2009.03.035 19398165

[3] Pascual M., Boix J., Felipo V., & Guerri C. (2009). Repeated alcohol administration during adolescence causes changes in the mesolimbic dopaminergic and glutamatergic systems and promotes alcohol intake in the adult rat. Journal of neurochemistry, 108(4), 920–931. 10.1111/j.1471-4159.2008.05835.x 19077056

[4] Brandon C. L., Marinelli M., Baker L. K., & White F. J. (2001). Enhanced reactivity and vulnerability to cocaine following methylphenidate treatment in adolescent rats. Neuropsychopharmacology, 25(5), 651–661. 10.1016/S0893-133X(01)00281-0 11682248

[5] Kroutil LA, Van Brunt DL, Herman-Stahl MA, Heller DC, Bray RM, Penne MA. Nonmedical use of prescription stimulants in the United States. Drug and alcohol dependence. 2006 9 15;84(2):135–43. 10.1016/j.drugalcdep.2005.12.011 16480836

[6] Office of Disease Prevention and Health Promotion. US Department of Health and Human Services: Healthy People 2010. http://www/health/gov/healthypeople/. 2000.

[7] Anker JJ, Carroll ME. Reinstatement of cocaine seeking induced by drugs, cues, and stress in adolescent and adult rats. Psychopharmacology. 2010 2 1;208(2):211–22. 10.1007/s00213-009-1721-2 19953228 PMC3228249

[8] Brenhouse HC, Andersen SL. Delayed extinction and stronger reinstatement of cocaine conditioned place preference in adolescent rats, compared to adults. Behavioral neuroscience. 2008 4;122(2):460. 10.1037/0735-7044.122.2.460 18410184 PMC5267226

[9] Chambers RA, Taylor JR, Potenza MN. Developmental neurocircuitry of motivation in adolescence: a critical period of addiction vulnerability. American Journal of Psychiatry. 2003 6 1;160(6):1041–52 10.1176/appi.ajp.160.6.1041 12777258 PMC2919168

[10] Edwards S, Koob GF. Experimental psychiatric illness and drug abuse models: from human to animal, an overview. Psychiatric Disorders 2012 (31–48). Humana Press. 10.1007/978-1-61779-458-2_2 22231805 PMC3285446

[11] Crews FT, Walter TJ, Coleman LG, Vetreno RP. Toll-like receptor signaling and stages of addiction. Psychopharmacology. 2017 5 1;234(9–10):1483–98. 10.1007/s00213-017-4560-6 28210782 PMC5420377

[12] Guerri C, Pascual M. Impact of neuroimmune activation induced by alcohol or drug abuse on adolescent brain development. International Journal of Developmental Neuroscience. 2019 10 1;77:89–98. 10.1016/j.ijdevneu.2018.11.006 30468786

[13] Brenhouse HC, Schwarz JM. Immunoadolescence: neuroimmune development and adolescent behavior. Neuroscience & Biobehavioral Reviews. 2016 11 1;70:288–99. 10.1016/j.neubiorev.2016.05.035 27260127 PMC5412135

[14] Sanchez-Alavez M, Nguyen W, Mori S, Wills DN, Otero D, Ehlers CL, et al. Time course of microglia activation and brain and blood cytokine/chemokine levels following chronic ethanol exposure and protracted withdrawal in rats. Alcohol. 2019 5 1;76:37–45. 10.1016/j.alcohol.2018.07.005 30554034 PMC6336521

[15] Schneider R, Bandiera S, Souza DG, Bellaver B, Caletti G, Quincozes-Santos A et al. N-acetylcysteine prevents alcohol related neuroinflammation in rats. Neurochemical research. 2017 8 1;42(8):2135–41. 10.1007/s11064-017-2218-8 28303497

[16] El‐Hage N, Gurwell JA, Singh IN, Knapp PE, Nath A, Hauser KF. Synergistic increases in intracellular Ca2+, and the release of MCP‐1, RANTES, and IL‐6 by astrocytes treated with opiates and HIV‐1 Tat. Glia. 2005 4 15;50(2):91–106. 10.1002/glia.20148 15630704 PMC4301446

[17] Hutchinson MR, Coats BD, Lewis SS, Zhang Y, Sprunger DB, Rezvani N, et al. Proinflammatory cytokines oppose opioid-induced acute and chronic analgesia. Brain, behavior, and immunity. 2008 11 1;22(8):1178–89. 10.1016/j.bbi.2008.05.004 18599265 PMC2783238

[18] Piotrowska A, Rojewska E, Pawlik K, Kreiner G, Ciechanowska A, Makuch W, et al. Pharmacological blockade of CXCR3 by (±)-NBI-74330 reduces neuropathic pain and enhances opioid effectiveness-evidence from in vivo and in vitro studies. Biochimica et Biophysica Acta (BBA)-Molecular Basis of Disease. 2018 10 1;1864(10):3418–37. 10.1016/j.bbadis.2018.07.032 30076959

[19] Wang W, Peng Y, Yang H, Bu H, Guo G, Liu D, et al. Potential role of CXCL10/CXCR3 signaling in the development of morphine tolerance in periaqueductal gray. Neuropeptides. 2017 10 1;65:120–7. 10.1016/j.npep.2017.07.004 28755808

[20] Bezzi P, Domercq M, Brambilla L, Galli R, Schols D, De Clercq E, et al. CXCR4-activated astrocyte glutamate release via TNFα: amplification by microglia triggers neurotoxicity. Nature neuroscience. 2001 7;4(7):702–10. 10.1038/89490 11426226

[21] Guyon A, Nahon JL. Multiple actions of the chemokine stromal cell-derived factor-1α on neuronal activity. Journal of molecular endocrinology. 2007 3 1;38(3):365–76. 10.1677/JME-06-0013 17339399

[22] Endres MJ, Clapham PR, Marsh M, Ahuja M, Turner JD, McKnight A, et al. CD4-independent infection by HIV-2 is mediated by fusin/CXCR4. Cell. 1996 11 15;87(4):745–56 10.1016/s0092-8674(00)81393-8 8929542

[23] Zhang L, Huang Y, He T, Cao Y, Ho DD. HIV-1 subtype and second-receptor use. Nature. 1996 10;383(6603):768–. 10.1038/383768a0 8892998

[24] Peng H, Huang Y, Rose J, Erichsen D, Herek S, Fujii N, et al. Stromal cell‐derived factor 1‐mediated CXCR4 signaling in rat and human cortical neural progenitor cells. Journal of neuroscience research. 2004 4 1;76(1):35–50. 10.1002/jnr.20045 15048928

[25] Yang F, Sun W, Yang Y, Wang Y, Li CL, Fu H, et al. SDF1–CXCR4 signaling contributes to persistent pain and hypersensitivity via regulating excitability of primary nociceptive neurons: involvement of ERK-dependent Nav1. 8 up-regulation. Journal of neuroinflammation. 2015 12 1;12(1):219. 10.1186/s12974-015-0441-2 26597700 PMC4657286

[26] Li H, Wang R. A focus on CXCR4 in Alzheimer’s disease. Brain Circulation. 2017 10;3(4):199. 10.4103/bc.bc_13_17 30276325 PMC6057706

[27] Ehtesham M, Stevenson CB, Thompson RC. Preferential expression of chemokine receptor CXCR4 by highly malignant human gliomas and its association with poor patient survival. Neurosurgery. 2008 10 1;63(4):E820. 10.1227/01.NEU.0000325687.45344.9E 18981857

[28] Tseng D, Vasquez-Medrano DA, Brown JM. Targeting SDF-1/CXCR4 to inhibit tumour vasculature for treatment of glioblastomas. British journal of cancer. 2011 6;104(12):1805. 10.1038/bjc.2011.169 21587260 PMC3111201

[29] Rocha NP, de Miranda AS, Teixeira AL. Insights into neuroinflammation in Parkinson’s disease: from biomarkers to anti-inflammatory based therapies. BioMed research international. 2015 10;2015. 10.1155/2015/628192 26295044 PMC4532803

[30] Xia M, Hyman BT. Chemokines/chemokine receptors in the central nervous system and Alzheimer’s disease. Journal of neurovirology. 1999 1 1;5(1):32–41. 10.3109/13550289909029743 10190688

[31] Werner L, Guzner-Gur H, Dotan I. Involvement of CXCR4/CXCR7/CXCL12 Interactions in Inflammatory bowel disease. Theranostics. 2013;3(1):40. 10.7150/thno.5135 23382785 PMC3563080

[32] Chu PY, Zatta A, Kiriazis H, Chin-Dusting J, Du XJ, Marshall T, et al. CXCR4 antagonism attenuates the cardiorenal consequences of mineralocorticoid excess. Circulation: Heart Failure. 2011 9;4(5):651–8. 10.1161/CIRCHEARTFAILURE.110.960831 21685249

[33] Li JK, Yu L, Shen Y, Zhou LS, Wang YC, Zhang JH. Inhibition of CXCR4 activity with AMD3100 decreases invasion of human colorectal cancer cells in vitro. World Journal of Gastroenterology. 2008 4 21;14(15):2308. 10.3748/wjg.14.2308 18416455 PMC2705083

[34] Das S, Basu A. Inflammation: a new candidate in modulating adult neurogenesis. Journal of neuroscience research. 2008 5 1;86(6):1199–208. 10.1002/jnr.21585 18058947

[35] Hermann GE, Van Meter MJ, Rogers RC. CXCR4 receptors in the dorsal medulla: implications for autonomic dysfunction. European Journal of Neuroscience. 2008 2;27(4):855–64. 10.1111/j.1460-9568.2008.06058.x 18333961 PMC3951345

[36] Oliver CF, Simmons SJ, Nayak SU, Smith GR, Reitz AB, Rawls SM. Chemokines and ‘bath salts’: CXCR4 receptor antagonist reduces rewarding and locomotor-stimulant effects of the designer cathinone MDPV in rats. Drug and Alcohol Dependence. 2018 5 1;186:75–9. 10.1016/j.drugalcdep.2018.01.013 29550625 PMC5911211

[37] Kim J, Connelly KL, Unterwald EM, Rawls SM. Chemokines and cocaine: CXCR4 receptor antagonist AMD3100 attenuates cocaine place preference and locomotor stimulation in rats. Brain, Behavior, and Immunity. 2017 5 1;62:30–4. 10.1016/j.bbi.2016.08.015 27575003 PMC5326690

[38] Araos P, Pedraz M, Serrano A, Lucena M, Barrios V, García‐Marchena N, et al. Plasma profile of pro‐inflammatory cytokines and chemokines in cocaine users under outpatient treatment: influence of cocaine symptom severity and psychiatric co‐morbidity. Addiction Biology. 2015 7;20(4):756–72. 10.1111/adb.12156 24854157

[39] Berríos-Cárcamo P, Quezada M, Quintanilla ME, Morales P, Ezquer M, Herrera-Marschitz M, et al. Oxidative Stress and Neuroinflammation as a Pivot in Drug Abuse. A Focus on the Therapeutic Potential of Antioxidant and Anti-Inflammatory Agents and Biomolecules. Antioxidants. 2020 9;9(9):830. 10.3390/antiox9090830 32899889 PMC7555323

[40] Ferrer-Pérez C, Martinez TE, Montagud-Romero S, Ballestín R, Reguilón MD, Miñarro J, et al. Indomethacin blocks the increased conditioned rewarding effects of cocaine induced by repeated social defeat. PLoS One. 2018 12 17;13(12):e0209291. 10.1371/journal.pone.0209291 30557308 PMC6296503

[41] Ciccarone D. Stimulant abuse: pharmacology, cocaine, methamphetamine, treatment, attempts at pharmacotherapy. Primary Care: Clinics in Office Practice. 2011 3 1;38(1):41–58. 10.1016/j.pop.2010.11.004 21356420 PMC3056348

[42] Vrana SL, Vrana KE, Koves TR, Smith JE, Dworkin SI. Chronic cocaine administration increases CNS tyrosine hydroxylase enzyme activity and mRNA levels and tryptophan hydroxylase enzyme activity levels. Journal of Neurochemistry. 1993 12;61(6):2262–8. 10.1111/j.1471-4159.1993.tb07468.x 7902421

[43] Chiang YC, Chen PC, Chen JC. D3 dopamine receptors are down-regulated in amphetamine sensitized rats and their putative antagonists modulate the locomotor sensitization to amphetamine. Brain Research. 2003 5 16;972(1–2):159–67. 10.1016/s0006-8993(03)02522-8 12711089

[44] Ashok AH, Mizuno Y, Volkow ND, Howes OD. Association of stimulant use with dopaminergic alterations in users of cocaine, amphetamine, or methamphetamine: a systematic review and meta-analysis. JAMA psychiatry. 2017 5 1;74(5):511–9. 10.1001/jamapsychiatry.2017.0135 28297025 PMC5419581

[45] Wise RA. Drug-activation of brain reward pathways. Drug and alcohol dependence. 1998 6 1;51(1–2):13–22. 10.1016/s0376-8716(98)00063-5 9716927

[46] Huang Q, Zhou D, Chase K, Gusella JF, Aronin N, DiFiglia M. Immunohistochemical localization of the D1 dopamine receptor in rat brain reveals its axonal transport, pre-and postsynaptic localization, and prevalence in the basal ganglia, limbic system, and thalamic reticular nucleus. Proceedings of the National Academy of Sciences. 1992 12 15;89(24):11988–92. 10.1073/pnas.89.24.11988 PMC50683 1281547

[47] Hall H, Sedvall G, Magnusson O, Kopp J, Halldin C, Farde L. Distribution of D 1-and D 2-dopamine receptors, and dopamine and its metabolites in the human brain. Neuropsychopharmacology. 1994 12;11(4):245–56. 10.1038/sj.npp.1380111 7531978

[48] Woolverton WL, Virus RM. The effects of a D1 and a D2 dopamine antagonist on behavior maintained by cocaine or food. Pharmacology Biochemistry and Behavior. 1989 3 1;32(3):691–7. 10.1016/0091-3057(89)90019-1 2662223

[49] Robinson TE, Berridge KC. Incentive‐sensitization and addiction. Addiction. 2001 1;96(1):103–14. 10.1046/j.1360-0443.2001.9611038.x 11177523

[50] Yokel RA, Wise RA. Attenuation of intravenous amphetamine reinforcement by central dopamine blockade in rats. Psychopharmacology. 1976 1 1;48(3):311–8. 10.1007/BF00496868 823588

[51] Tham TN, Lazarini F, Franceschini IA, Lachapelle F, Amara A, Dubois‐Dalcq M. Developmental pattern of expression of the alpha chemokine stromal cell‐derived factor 1 in the rat central nervous system. European Journal of Neuroscience. 2001 3;13(5):845–56. 10.1046/j.0953-816x.2000.01451.x 11264658

[52] Van der Meer P, Ulrich AM, Gonźalez-Scarano F, Lavi E. Immunohistochemical analysis of CCR2, CCR3, CCR5, and CXCR4 in the human brain: potential mechanisms for HIV dementia. Experimental and Molecular Pathology. 2000 12 1;69(3):192–201. 10.1006/exmp.2000.2336 11115360

[53] Clark KH, Wiley CA, Bradberry CW. Psychostimulant abuse and neuroinflammation: emerging evidence of their interconnection. Neurotoxicity research. 2013 2 1;23(2):174–88. 10.1007/s12640-012-9334-7 22714667

[54] Yamamoto BK, Moszczynska A, Gudelsky GA. Amphetamine toxicities Classical and emerging mechanisms. Annals of the New York Academy of Sciences. 2010 2;1187:101. 10.1111/j.1749-6632.2009.05141.x 20201848 PMC3955986

[55] Lieberknecht V, Junqueira SC, Cunha MP, Barbosa TA, de Souza LF, Coelho IS, et al. Pramipexole, a dopamine D2/D3 receptor-preferring agonist, prevents experimental autoimmune encephalomyelitis development in mice. Molecular neurobiology. 2017 3 1;54(2):1033–45. 10.1007/s12035-016-9717-5 26801190

[56] Pacheco R, Contreras F, Zouali M. The dopaminergic system in autoimmune diseases. Frontiers in immunology. 2014 3 21;5:117. 10.3389/fimmu.2014.00117 24711809 PMC3968755

[57] Zhang X, Liu T, Zhou Z, Mu X, Song C, Xiao T, et al. Enriched environment altered aberrant hippocampal neurogenesis and improved long-term consequences after temporal lobe epilepsy in adult rats. Journal of Molecular Neuroscience. 2015 6 1;56(2):409–21. 10.1007/s12031-015-0571-0 25946980

[58] Klein RS, Rubin JB. Immune and nervous system CXCL12 and CXCR4: parallel roles in patterning and plasticity. Trends in immunology. 2004 6 1;25(6):306–14. 10.1016/j.it.2004.04.002 15145320

[59] Ahmed F, Tessarollo L, Thiele C, Mocchetti I. Brain-derived neurotrophic factor modulates expression of chemokine receptors in the brain. Brain Research. 2008 8 28;1227:1–1. 10.1016/j.brainres.2008.05.086 18588860 PMC2601566

[60] Azoulay D, Herishanu Y, Shapiro M, Brandshaft Y, Suriu C, Akria L, et al. Elevated serum BDNF levels are associated with favorable outcome in CLL patients: possible link to CXCR4 downregulation. Experimental Hematology. 2018 7 1;63:17–21. 10.1016/j.exphem.2018.04.005 29705266

[61] Abe P, Wüst HM, Arnold SJ, van de Pavert SA, Stumm R. CXCL12‐mediated feedback from granule neurons regulates generation and positioning of new neurons in the dentate gyrus. Glia. 2018 8;66(8):1566–76. 10.1002/glia.23324 29537098

[62] Floresco SB, Blaha CD, Yang CR, Phillips AG. Modulation of hippocampal and amygdalar-evoked activity of nucleus accumbens neurons by dopamine: cellular mechanisms of input selection. Journal of Neuroscience. 2001 4 15;21(8):2851–60. 10.1523/JNEUROSCI.21-08-02851.2001 11306637 PMC6762526

[63] Goto Y, Grace AA. Dopaminergic modulation of limbic and cortical drive of nucleus accumbens in goal-directed behavior. Nature neuroscience. 2005 6;8(6):805–12. 10.1038/nn1471 15908948

[64] Li G, Adesnik H, Li J, Long J, Nicoll RA, Rubenstein JL, et al. Regional distribution of cortical interneurons and development of inhibitory tone are regulated by Cxcl12/Cxcr4 signaling. Journal of Neuroscience. 2008 1 30;28(5):1085–98. 10.1523/JNEUROSCI.4602-07.2008 18234887 PMC3072297

[65] Tiveron MC, Rossel M, Moepps B, Zhang YL, Seidenfaden R, Favor J, et al. Molecular interaction between projection neuron precursors and invading interneurons via stromal-derived factor 1 (CXCL12)/CXCR4 signaling in the cortical subventricular zone/intermediate zone. Journal of Neuroscience. 2006 12 20;26(51):13273–8. 10.1523/JNEUROSCI.4162-06.2006 17182777 PMC6674999

[66] Stumm RK, Zhou C, Ara T, Lazarini F, Dubois-Dalcq M, Nagasawa T, et al. CXCR4 regulates interneuron migration in the developing neocortex. Journal of Neuroscience. 2003 6 15;23(12):5123–30. 10.1523/JNEUROSCI.23-12-05123.2003 12832536 PMC6741192

[67] Ni HT, Hu S, Sheng WS, Olson JM, Cheeran MC, Chan AS, et al. High-level expression of functional chemokine receptor CXCR4 on human neural precursor cells. Developmental Brain Research. 2004 9 17;152(2):159–69. 10.1016/j.devbrainres.2004.06.015 15351504

[68] Sillivan SE, Black YD, Naydenov AV, Vassoler FR, Hanlin RP, Konradi C. Binge cocaine administration in adolescent rats affects amygdalar gene expression patterns and alters anxiety-related behavior in adulthood. Biological Psychiatry. 2011 9 15;70(6):583–92. 10.1016/j.biopsych.2011.03.035 21571252 PMC3159046

[69] Faul F, Erdfelder E, Lang AG, Buchner A. G* Power 3: A flexible statistical power analysis program for the social, behavioral, and biomedical sciences. Behavior Research Methods. 2007 5 1;39(2):175–91. 10.3758/bf03193146 17695343

[70] Ravenelle R, Santolucito HB, Byrnes EM, Byrnes JJ, Donaldson ST. Housing environment modulates physiological and behavioral responses to anxiogenic stimuli in trait anxiety male rats. Neuroscience. 2014 6 13;270:76–87. 10.1016/j.neuroscience.2014.03.060 24713371 PMC4047719

[71] Mason B, Rollins LG, Asumadu E, Cange C, Walton N, Donaldson ST. Nesting environment provides sex-specific neuroprotection in a rat model of neonatal hypoxic-ischemic injury. Frontiers in behavioral neuroscience. 2018 10 2;12:221. 10.3389/fnbeh.2018.00221 30356904 PMC6190890

[72] Ravenelle R, Byrnes EM, Byrnes JJ, McInnis C, Park JH, Donaldson ST. Environmental enrichment effects on the neurobehavioral profile of selective outbred trait anxiety rats. Behavioral Brain Research. 2013 9 1;252:49–57. 10.1016/j.bbr.2013.05.041 23727174 PMC4256945

[73] Paxinos G., & Watson C. (2006). The rat brain in stereotaxic coordinates: hard cover edition. Elsevier. 10.1016/0165-0270(80)90021-7 6110810

[74] Garcia-Segura LM, Perez-Marquez J. A new mathematical function to evaluate neuronal morphology using the Sholl analysis. Journal of Neuroscience Methods. 2014 4 15;226:103–9. 10.1016/j.jneumeth.2014.01.016 24503022

[75] Cholanian M, Lobzova A, Das B, Yelleswarapu C, Donaldson ST. Digital holographic microscopy discriminates sex differences in medial prefrontal cortex GABA neurons following amphetamine sensitization. Pharmacology Biochemistry and Behavior. 2014 9 1;124:326–32. 10.1016/j.pbb.2014.06.026 24999221

[76] Basova L, Najera JA, Bortell N, Wang D, Moya R, Lindsey A, et al. Dopamine and its receptors play a role in the modulation of CCR5 expression in innate immune cells following exposure to methamphetamine: implications to HIV infection. PloS One. 2018 6 26;13(6):e0199861. 10.1371/journal.pone.0199861 29944719 PMC6019408

[77] Nayak SU, Cicalese S, Tallarida C, Oliver CF, Rawls SM. Chemokine CCR5 and cocaine interactions in the brain: Cocaine enhances mesolimbic CCR5 mRNA levels and produces place preference and locomotor activation that are reduced by a CCR5 antagonist. Brain, Behavior, and Immunity. 2020 1 1;83:288–92. 10.1016/j.bbi.2019.09.017 31557508 PMC6906231

[78] Wakida N, Kiguchi N, Saika F, Nishiue H, Kobayashi Y, Kishioka S. CC-chemokine ligand 2 facilitates conditioned place preference to methamphetamine through the activation of dopamine systems. Journal of Pharmacological Sciences. 2014 5 20;125(1):68–73. 10.1254/jphs.14032fp 24748435

[79] Ikegami D, Narita M, Imai S, Miyashita K, Tamura R, Narita M, et al. PRECLINICAL STUDY: BRIEF REPORT: Epigenetic modulation at the CCR2 gene correlates with the maintenance of behavioral sensitization to methamphetamine. Addiction Biology. 2010 7;15(3):358–61. 10.1111/j.1369-1600.2010.00219.x 20624155

[80] Banisadr G, Fontanges P, Haour F, Kitabgi P, Rostène W, Mélik Parsadaniantz S. Neuroanatomical distribution of CXCR4 in adult rat brain and its localization in cholinergic and dopaminergic neurons. European Journal of Neuroscience. 2002 11;16(9):1661–71. 10.1046/j.1460-9568.2002.02237.x 12431218

[81] Trecki J, Brailoiu GC, Unterwald EM. Localization of CXCR4 in the forebrain of the adult rat. Brain Research. 2010 2 22;1315:53–62. 10.1016/j.brainres.2009.12.015 20026091 PMC2826800

[82] Peterson JD, Wolf ME, White FJ. Altered responsiveness of medial prefrontal cortex neurons to glutamate and dopamine after withdrawal from repeated amphetamine treatment. Synapse. 2000 6 15;36(4):342–4. 10.1002/(SICI)1098-2396(20000615)36:4&lt;342::AID-SYN11&gt;3.0.CO;2-9 10819912

[83] Hedou G, Homberg J, Feldon J, Heidbreder CA. Expression of sensitization to amphetamine and dynamics of dopamine neurotransmission in different laminae of the rat medial prefrontal cortex. Neuropharmacology. 2001 3 1;40(3):366–82. 10.1016/s0028-3908(00)00174-x 11166330

[84] Koss WA, Belden CE, Hristov AD, Juraska JM. Dendritic remodeling in the adolescent medial prefrontal cortex and the basolateral amygdala of male and female rats. Synapse. 2014 2;68(2):61–72. 10.1002/syn.21716 24105875

[85] Willing J, Juraska JM. The timing of neuronal loss across adolescence in the medial prefrontal cortex of male and female rats. Neuroscience. 2015 8 20;301:268–75. 10.1016/j.neuroscience.2015.05.073 26047728 PMC4504753

[86] Kalsbeek A, Voorn P, Buijs RM, Pool CW, Uylings HB. Development of the dopaminergic innervation in the prefrontal cortex of the rat. Journal of comparative neurology. 1988 3 1;269(1):58–72. 10.1002/cne.902690105 3361004

[87] Tseng KY, O’Donnell P. Dopamine modulation of prefrontal cortical interneurons changes during adolescence. Cerebral Cortex. 2007 5 1;17(5):1235–40. 10.1093/cercor/bhl034 16818475 PMC2204087

[88] Galvan A, Hare TA, Parra CE, Penn J, Voss H, Glover G, et al. Earlier development of the accumbens relative to orbitofrontal cortex might underlie risk-taking behavior in adolescents. Journal of Neuroscience. 2006 6 21;26(25):6885–92. 10.1523/JNEUROSCI.1062-06.2006 16793895 PMC6673830

[89] Eshel N, Nelson EE, Blair RJ, Pine DS, Ernst M. Neural substrates of choice selection in adults and adolescents: development of the ventrolateral prefrontal and anterior cingulate cortices. Neuropsychologia. 2007 1 1;45(6):1270–9. 10.1016/j.neuropsychologia.2006.10.004 17118409 PMC2700731

[90] Somerville LH, Hare T, Casey BJ. Frontostriatal maturation predicts cognitive control failure to appetitive cues in adolescents. Journal of cognitive neuroscience. 2011 9;23(9):2123–34. 10.1162/jocn.2010.21572 20809855 PMC3131482

[91] Knudsen EI. Sensitive periods in the development of the brain and behavior. Journal of cognitive neuroscience. 2004 10;16(8):1412–25. 10.1162/0898929042304796 15509387

[92] Van den Bos W, Cohen MX, Kahnt T, Crone EA. Striatum–medial prefrontal cortex connectivity predicts developmental changes in reinforcement learning. Cerebral cortex. 2012 6 1;22(6):1247–55. 10.1093/cercor/bhr198 21817091 PMC6283353

[93] Kang S, Wu MM, Galvez R, Gulley JM. Timing of amphetamine exposure in relation to puberty onset determines its effects on anhedonia, exploratory behavior, and dopamine D1 receptor expression in young adulthood. Neuroscience. 2016 12 17;339:72–84. 10.1016/j.neuroscience.2016.09.044 27702645 PMC5118128

[94] Ruskin DN, Marshall JF. Amphetamine‐and cocaine‐induced fos in the rat striatum depends on D2 dopamine receptor activation. Synapse. 1994 11;18(3):233–40. 10.1002/syn.890180309 7855736

[95] Hammerslag LR, Waldman AJ, Gulley JM. Effects of amphetamine exposure in adolescence or young adulthood on inhibitory control in adult male and female rats. Behavioural brain research. 2014 4 15;263:22–33. 10.1016/j.bbr.2014.01.015 24462963 PMC3954703

[96] Hankosky ER, Gulley JM. Performance on an impulse control task is altered in adult rats exposed to amphetamine during adolescence. Developmental psychobiology. 2013 11;55(7):733–44. 10.1002/dev.21067 PMC3491181 22778047

[97] Hankosky ER, Kofsky NM, Gulley JM. Age of exposure-dependent effects of amphetamine on behavioral flexibility. Behavioural brain research. 2013 9 1;252:117–25. 10.1016/j.bbr.2013.06.002 23756139 PMC3742571

[98] Westbrook SR, Dwyer MR, Cortes LR, Gulley JM. Extended access self-administration of methamphetamine is associated with age-and sex-dependent differences in drug taking behavior and recognition memory in rats. Behavioural Brain Research. 2020 5 8:112659. 10.1016/j.bbr.2020.112659 32437887 PMC7307427

[99] Spear LP, Brake SC. Periadolescence: age‐dependent behavior and psychopharmacological responsivity in rats. Developmental Psychobiology: The Journal of the International Society for Developmental Psychobiology. 1983 3;16(2):83–109. 10.1002/dev.420160203 6339302

[100] Andersen SL. Stimulants and the developing brain. Trends in Pharmacological Sciences. 2005 5 1;26(5):237–43. 10.1016/j.tips.2005.03.009 15860370

[101] Lynch WJ, Carroll ME. Sex differences in the acquisition of intravenously self-administered cocaine and heroin in rats. Psychopharmacology. 1999 5 1;144(1):77–82. 10.1007/s002130050979 10379627

[102] Lynch WJ, Roth ME, Carroll ME. Biological basis of sex differences in drug abuse: preclinical and clinical studies. Psychopharmacology. 2002 11 1;164(2):121–37. 10.1007/s00213-002-1183-2 12404074

[103] Pierce R. C., & Kalivas P. W. (1997). A circuitry model of the expression of behavioral sensitization to amphetamine-like psychostimulants. *Brain research reviews*, 25(2), 192–216. 10.1016/s0165-0173(97)00021-0 9403138

[104] Kabbaj M., Isgor C., Watson S. J., & Akil H. (2002). Stress during adolescence alters behavioral sensitization to amphetamine. *Neuroscience*, 113(2), 395–400. 10.1016/s0306-4522(02)00188-4 12127096

[105] Rivet J. M., Stinus L., LeMoal M., & Mormede P. (1989). Behavioral sensitization to amphetamine is dependent on corticosteroid receptor activation. *Brain research*, 498(1), 149–153. 10.1016/0006-8993(89)90411-3 2790466

[106] Caster J. M., Walker Q. D., & Kuhn C. M. (2007). A single high dose of cocaine induces differential sensitization to specific behaviors across adolescence. *Psychopharmacology*, 193(2), 247–260. 10.1007/s00213-007-0764-5 17426961

[107] Zou YR, Kottmann AH, Kuroda M, Taniuchi I, Littman DR. Function of the chemokine receptor CXCR4 in haematopoiesis and in cerebellar development. Nature. 1998 6;393(6685):595–9. 10.1038/31269 9634238

[108] Macht VA. Neuro-immune interactions across development: a look at glutamate in the prefrontal cortex. Neuroscience & Biobehavioral Reviews. 2016 12 1;71:267–80. 27593444 10.1016/j.neubiorev.2016.08.039

[109] Peng H, Kolb R, Kennedy JE, Zheng J. Differential expression of CXCL12 and CXCR4 during human fetal neural progenitor cell differentiation. Journal of Neuroimmune Pharmacology. 2007 9 1;2(3):251–8. 10.1007/s11481-007-9081-3 18040858 PMC2169289

[110] Stumm R, Höllt V. CXC chemokine receptor 4 regulates neuronal migration and axonal pathfinding in the developing nervous system: implications for neuronal regeneration in the adult brain. Journal of Molecular Endocrinology. 2007 3 1;38(3):377–82. 10.1677/JME-06-0032 17339400

[111] Borrell V, Marín O. Meninges control tangential migration of hem-derived Cajal-Retzius cells via CXCL12/CXCR4 signaling. Nature neuroscience. 2006 10;9(10):1284–93. 10.1038/nn1764 16964252

[112] Wong ML, Xin WW, Duman RS. Rat LCR1: cloning and cellular distribution of a putative chemokine receptor in brain. Molecular psychiatry. 1996 5 1;1(2):133–40. 9118323

[113] Chu J, Anderson SA. Development of cortical interneurons. Neuropsychopharmacology. 2015 1;40(1):16–23. 10.1038/npp.2014.171 25103177 PMC4262895

[114] Yang L, Wang M, Guo YY, Sun T, Li YJ, Yang Q, et al. Systemic inflammation induces anxiety disorder through CXCL12/CXCR4 pathway. Brain, behavior, and immunity. 2016 8 1;56:352–62. 10.1016/j.bbi.2016.03.001 26952745

[115] Boudot A, Kerdivel G, Habauzit D, Eeckhoute J, Le Dily F, Flouriot G, et al. Differential estrogen-regulation of CXCL12 chemokine receptors, CXCR4 and CXCR7, contributes to the growth effect of estrogens in breast cancer cells. PLOS ONE. 2011 6 10;6(6):e20898. 10.1371/journal.pone.0020898 21695171 PMC3112227

[116] Patterson BK, Czerniewski M, Andersson J, Sullivan Y, Su F, Jiyamapa D, et al. Regulation of CCR5 and CXCR4 expression by type 1 and type 2 cytokines: CCR5 expression is downregulated by IL-10 in CD4-positive lymphocytes. Clinical Immunology. 1999 6 1;91(3):254–62. 10.1006/clim.1999.4713 10370370

[117] Merkel SF, Razmpour R, Lutton EM, Tallarida CS, Heldt NA, Cannella LA, et al. Adolescent traumatic brain injury induces chronic mesolimbic neuroinflammation with concurrent enhancement in the rewarding effects of cocaine in mice during adulthood. Journal of neurotrauma. 2017 1 1;34(1):165–81. 10.1089/neu.2015.4275 27026056 PMC5198083

[118] Trocello JM, Rostene W, Melik-Parsadaniantz S, Godefroy D, Roze E, Kitabgi P, et al. Implication of CCR2 chemokine receptor in cocaine-induced sensitization. Journal of Molecular Neuroscience. 2011 7 1;44(3):147–51. 10.1007/s12031-011-9508-4 21424761

[119] Saika F, Kiguchi N, Wakida N, Kobayashi D, Fukazawa Y, Matsuzaki S, et al. Upregulation of CCL7 and CCL2 in reward system mediated through dopamine D1 receptor signaling underlies methamphetamine-induced place preference in mice. Neuroscience Letters. 2018 2 5;665:33–7. 10.1016/j.neulet.2017.11.042 29174638

[120] Trecki J, Unterwald EM. Modulation of cocaine-induced activity by intracerebral administration of CXCL12. Neuroscience. 2009 6 16;161(1):13–22. 10.1016/j.neuroscience.2009.03.027 19303923 PMC2680459

[121] Yang S, Edman LC, Sánchez-Alcañiz JA, Fritz N, Bonilla S, Hecht J, et al. Cxcl12/Cxcr4 signaling controls the migration and process orientation of A9-A10 dopaminergic neurons. Development. 2013 11 15;140(22):4554–64. 10.1242/dev.098145 24154522

[122] Wu PR, Cho KK, Vogt D, Sohal VS, Rubenstein JL. The cytokine CXCL12 promotes basket interneuron inhibitory synapses in the medial prefrontal cortex. Cerebral Cortex. 2017 9 1;27(9):4303–13. 10.1093/cercor/bhw230 27497284 PMC6410508

[123] Abe P, Molnár Z, Tzeng YS, Lai DM, Arnold SJ, Stumm R. Intermediate progenitors facilitate intracortical progression of thalamocortical axons and interneurons through CXCL12 chemokine signaling. Journal of Neuroscience. 2015 9 23;35(38):13053–63. 10.1523/JNEUROSCI.1488-15.2015 26400936 PMC6605439

[124] Singh AK, Arya RK, Trivedi AK, Sanyal S, Baral R, Dormond O, et al. Chemokine receptor trio: CXCR3, CXCR4 and CXCR7 crosstalk via CXCL11 and CXCL12. Cytokine & Growth Factor Reviews. 2013 2 1;24(1):41–9. 10.1016/j.cytogfr.2012.08.007 22989616 PMC4172454

[125] Levoye A, Balabanian K, Baleux F, Bachelerie F, Lagane B. CXCR7 heterodimerizes with CXCR4 and regulates CXCL12-mediated G protein signaling. Blood, The Journal of the American Society of Hematology. 2009 6 11;113(24):6085–93. 10.1182/blood-2008-12-196618 19380869

[126] Yaw AM, Prosser RA, Jones PC, Garcia BJ, Jacobson DA, Glass JD. Epigenetic effects of paternal cocaine on reward stimulus behavior and accumbens gene expression in mice. Behavioural Brain Research. 2019 7 23;367:68–81. 10.1016/j.bbr.2019.02.043 30910707

[127] Schwarz JM, Hutchinson MR, Bilbo SD. Early-life experience decreases drug-induced reinstatement of morphine CPP in adulthood via microglial-specific epigenetic programming of anti-inflammatory IL-10 expression. Journal of Neuroscience. 2011 12 7;31(49):17835–47. 10.1523/JNEUROSCI.3297-11.2011 22159099 PMC3259856

