# Supplementary figures and images for: CXCR4 inhibition with AMD3100 attenuates amphetamine induced locomotor activity in adolescent Long Evans male rats
Source: PLoS One. 2021 Mar 1;16(3):e0247707. doi: 10.1371/journal.pone.0247707 (PMC7920371; doi:10.1371/journal.pone.0247707)

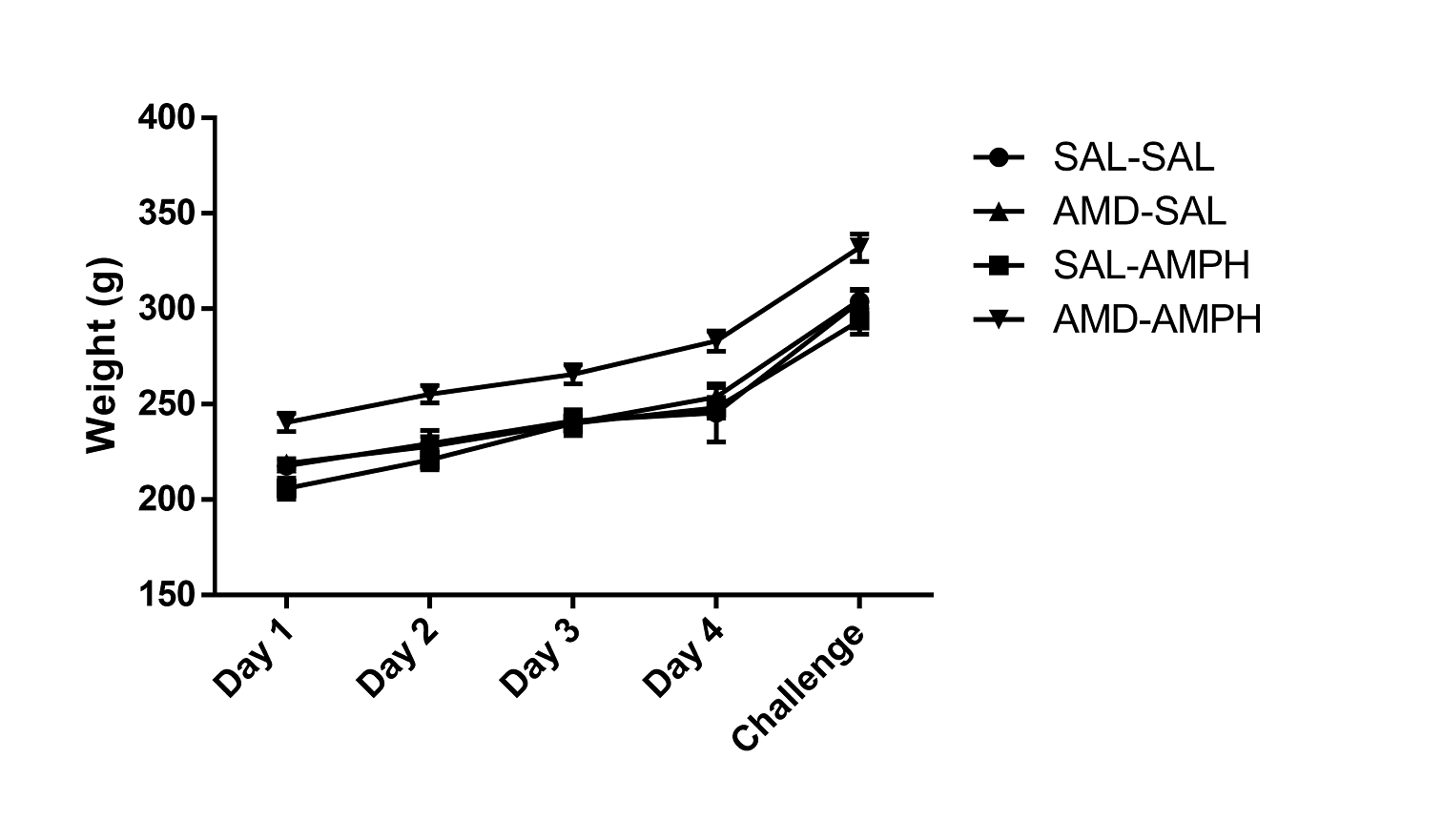

Supplement: S1 Fig — (TIF) [file pone.0247707.s001.tif]
